# Lightweight drone-deployed autonomous ocean profiler for repeated measurements in hazardous areas – Example from glacier fronts in NE Greenland

**DOI:** 10.1016/j.ohx.2022.e00313

**Published:** 2022-05-04

**Authors:** Ebbe Poulsen, Mathias Eggertsen, Erik H. Jepsen, Claus Melvad, Søren Rysgaard

**Affiliations:** a Department of Mechanical and Production Engineering, Aarhus University, Inge Lehmanns Gade 10, DK-8000 Aarhus C, Denmark; b Arctic Research Centre, Department of Biology, Aarhus University, Ole Worms Allé 1, DK-8000 Aarhus C, Denmark

**Keywords:** Autonomous profiler, Drone-deployment, Hazardous areas, Reusable CTD, Marine terminating glaciers, Arctic research

## Abstract

Accelerated melting of ice in Polar Regions due to global warming increases freshwater input to coastal waters from marine terminating glaciers. Lack of measurements near the glacier terminus limits our knowledge of the mixing processes between freshwater and the underlying ocean.

We present a low-cost (< € 3200) and lightweight (2.6 kg) drone-deployed, retrievable conductivity, temperature and depth (CTD) instrument for remote controlled (1 km) autonomous profiling in highly hazardous and remote areas. The instrument was deployed with a drone taking off from land and marine vessels to perform measurements near tidewater glaciers termini of the Greenland ice sheet.

The free-flowing profiler is reusable due to a compact ballast based single-shot buoyancy engine and post-profiling pickup by drone. It can reach a depth of up to 250 m, and is equipped with low-cost sensors for conductivity, temperature, and depth measurements.

During decent the profiler reaches a velocity of about 0.48 m/s, resulting in about 3.5 data points pr. m depth, but is designed to easily vary the velocity by changing buoyancy setup before deployment.

Successful tests were conducted at marine terminating glaciers in Northeast Greenland in August 2021.

## Graphical abstract

**Figure:**
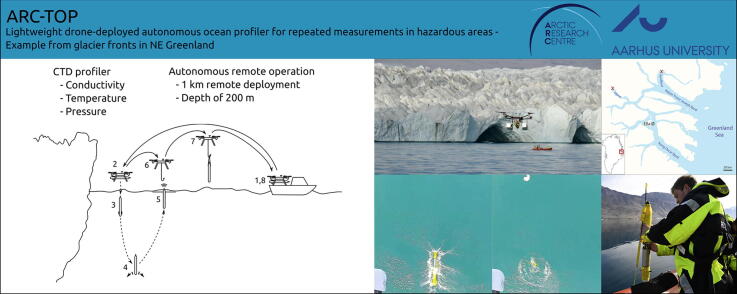

## Specifications table

**Table:** 

Hardware name	Arctic Research Centre Torpedo (ARC-TOP)
Subject area	Environmental, planetary and agricultural sciences
Hardware type	Field measurements and sensors
Closest commercial analog	Lockheed Martin XCTD Profiling System
Open source license	CC BY 4.0
Cost of hardware	€ 3200
Source file repository	https://doi.org/10.17632/zdvb5hzv2x.1

## Hardware in context

The rapid warming of the Planet has triggered an increasing interest in understanding melting of the Greenland ice sheet and its glaciers that floats into the ocean as it affects ocean circulation and the global sea level rise. At present it is not clear how warm ocean water reaches the tidewater glaciers or how and where meltwater is released into the fjord. Only few measurements in very close proximity to glacier termini have been made due to the risk of glacier calving. Previous measurement have used expendable CTD’s (XCTDs) from Sippican, Inc., deployed from helicopters [1], [2]. XCTDs are single-use profilers that decent at a known velocity, while transmitting measured conductivity and temperature to a receiver through wire. When the wire is fully extended, the wire breaks and the profiler is lost. Measurements by this method is severely limited by the cost of equipment replacement and helicopter hire.

The Jetyak, an unmanned surface vehicles (USV) with traditional CTDs have also been used previously [2], [3]. This remote controlled USV deploy a CTD by a winch system and represent a cost-effective alternative to XCTDs. However, this method is limited by access to the glacier termini when significant ice mélange is present.

The use of unmanned aerial vehicles (UAVs) to replace helicopters and USVs for CTD deployment have recently become viable options due to the rapid development of cheap and available hardware and software. A strict weight requirement is imposed on airborne equipment, why a low-cost, lightweight, and easy remote deployable instrument is needed to improve the economic and technical feasibility of glacier-ocean interaction studies.

## Hardware description

Evaluation of existing products and later development work revolved around the requirements outlined in Table 1. Given the highly specialized requirements, no commercial or open-source product were found to fulfil these to an acceptable degree.

**Table 1:** Product requirements and whether or not they are fulfilled by ARC-TOP.

	Product requirement	Value/specification	Achieved?
1. Usage	Deployment	From land and small marine vessel	Yes
	Handling	2 persons	Yes
	Operation	Autonomous profiling	Yes
	Reusability	> 50 deployments	Yes – Ballast is consumed
	Remote deployment distance	1 km	Yes
	Remote retrieve distance	1 km	Yes
	Communication	Wireless bi-directional transmission	Yes
	Broadcast position and health	1 km	No − 500 m achieved
2. Transport	Shipping	Fit on EUR-pallet (1200x800 mm)	Yes
	Battery	Below 100 Wh	Yes
	Weight	Max 15 kg	Yes
3. Environment	Air temperature	−25 to 30 °C	Yes
	Water temperature	−5 to 10 °C	Yes
	Wave tolerant	Calm weather, <1 m	Yes
	Ice tolerant	Operation in conditions with light ice mélange	Not tested
	Plume	Profiling within 10 m of SDG plume centre	Not tested
4. Design	Dimensions	Max 1100x200x200 mm	Yes
	Deployment weight	Max 5 kg	Yes – 2.6 kg
	Measured parameters	Conductivity (C), Temperature (T), Depth (D)^1^	Yes
	Measurement resolution	Min. 3 measurements per m	Yes
	Max depth	250 m	Yes
	Temperature acc.	± 0.1 °C	Yes
	Depth acc.	± 0.5 m	Yes
	Conductivity acc.	± 0.1 mS/cm	Yes
	Position acc.	± 10 m	Yes
	Price	Max € 4000	Yes
	Colour	High visibility	Yes

1 Depth, D, is a derived parameter from pressure, P, measurement using the equation: P=Dρg, where ρ is the water density and g is the gravitational constant. The current software does not correct for density variations due to changes in salinity.

**Fig. 1:**
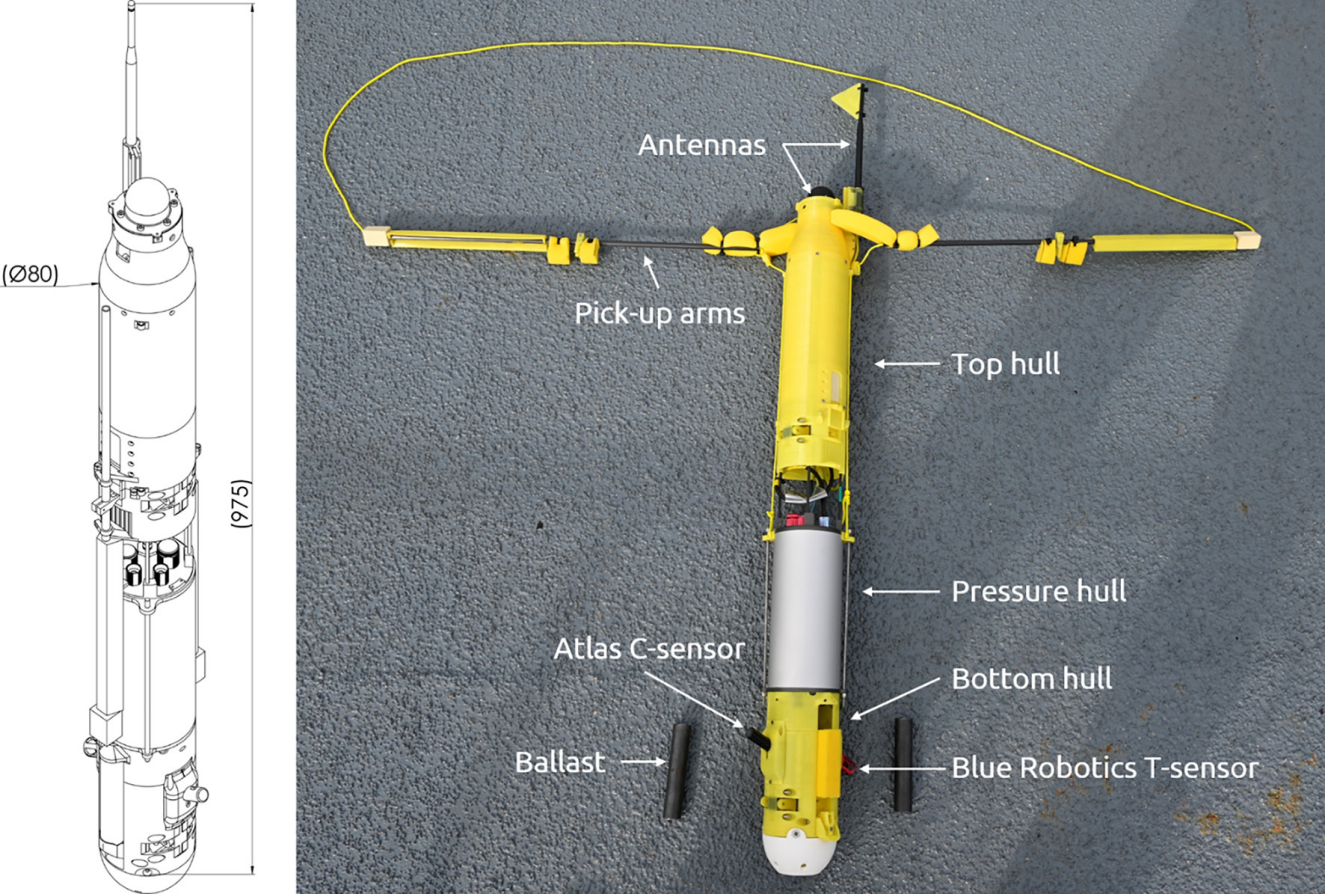
ARC-TOP with rough dimensions in mm (left) and the ARC-TOP with ballast and arms deployed (right). Photo courtesy of Peter Bondo Christensen.

**Fig. 2:**
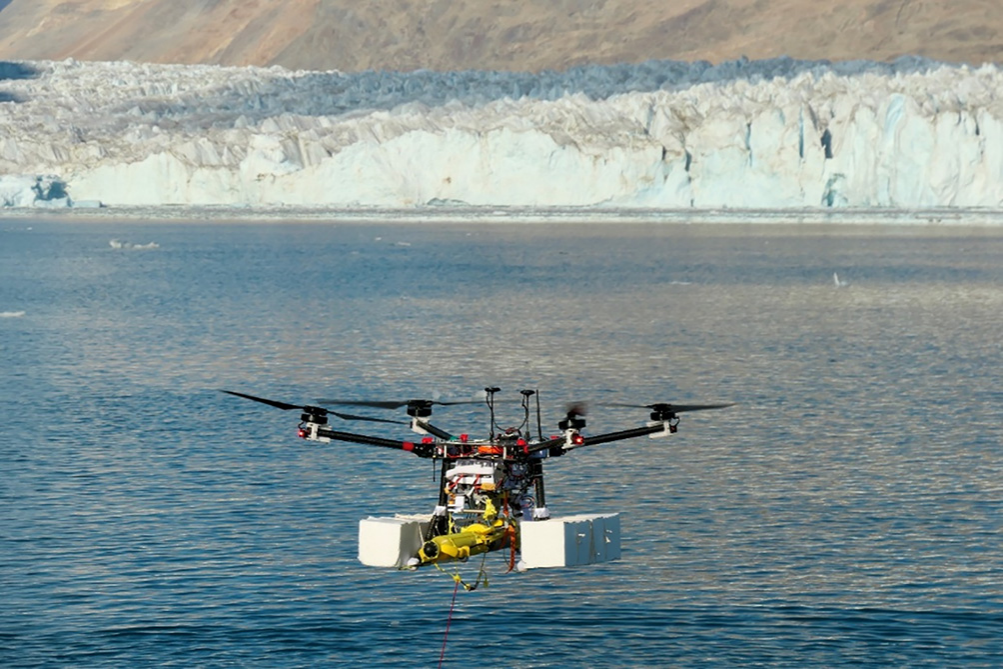
ARC-TOP mounted on UAV during testing in Isfjord in NE Greenland. Photo courtesy of Søren Rysgaard.

Development resulted in the ARC-TOP shown in Fig. 1 and Fig. 2, a compact and lightweight CTD with low-cost sensors, capable of UAV deployment and retrieval. The total weight ready for deployment is 2.6 kg. To reduce costs, the use of machined parts were kept at an absolute minimum. This led to a flooded design, where only the bare minimum of components are located inside the aluminium pressure hull, while the majority of the structure consist of 3D-printed sections for flow optimization. This reduces the size and weight of the profiler, with an increase in reparability, ease of configuration for other sensors/use-cases and speed of development.

**Fig. 3:**
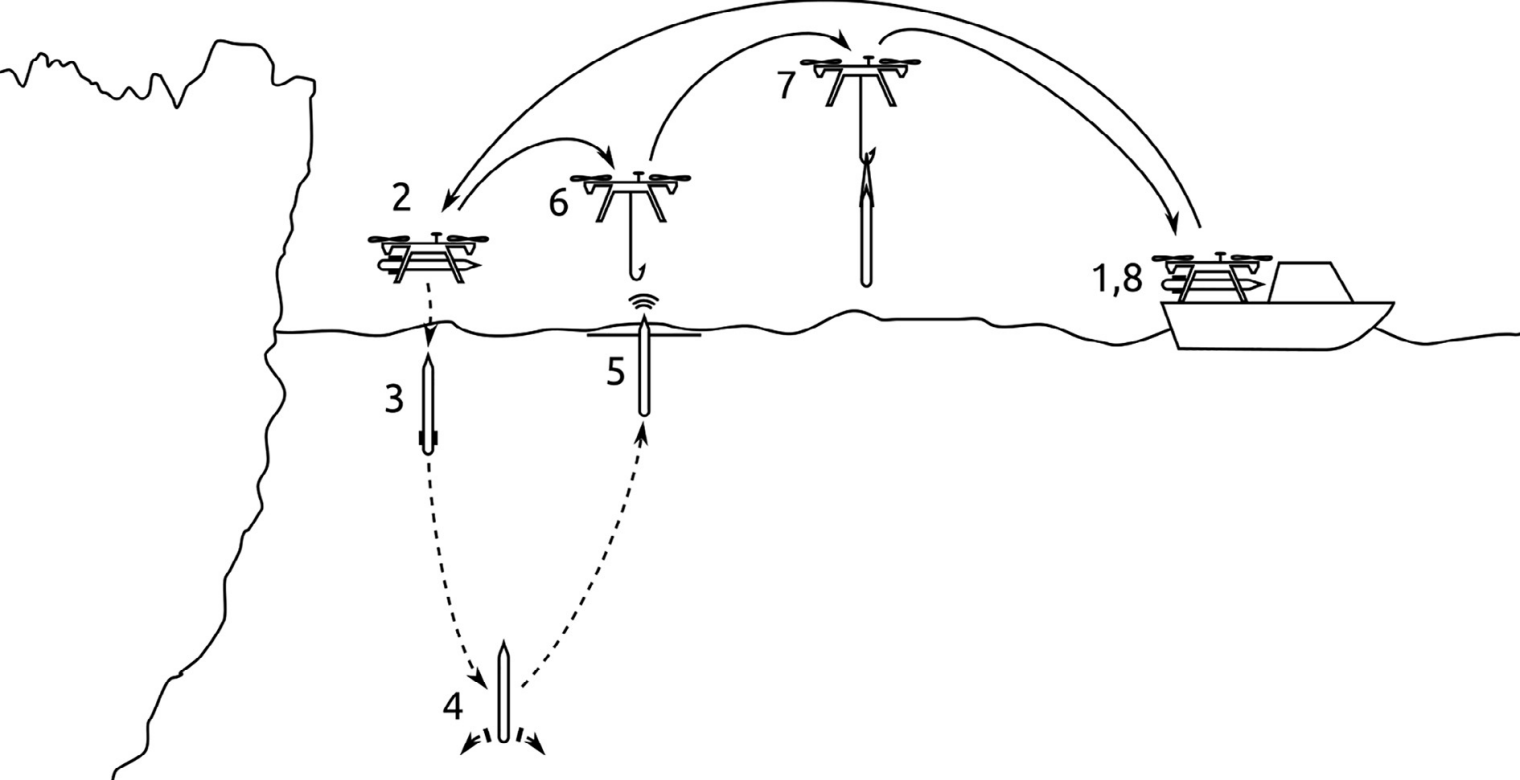
Operational concept of ARC-TOP consisting of setup and arming (1) flight to deployment position at glacier termini (2) drop and dive to set depth (3) ballast release and ascent (4) deployment of pick-up arms and position broadcast at surface (5) pick-up by UAV (6) return flight (7) and instrument retrieval and disarming (8).

The profiler is designed to be mounted underneath any suitable size UAV with available connections for three external servos. In hover at low altitude (≈ 1 m), the profiler is dropped, and upon submerging, begins autonomous operation. Due to the use of a ballast-based buoyancy engine, the profiler descends while logging conductivity, temperature and pressure. At a user configurable depth the ballast is dropped and the profiler thus achieves net positive buoyancy, resulting in ascent. Upon returning to the surface, the profiler broadcasts its position and deploys arms for subsequent pick-up. See Fig. 3 for an operating concept visualization.

Main advantages of the ARC-TOP design is:

• Ocean profiling in hazardous areas fx
  o Close proximity to glacier termini
  o Waters with high risk of marine vessels running aground
  o Inaccessibility due to terrain
• Cost-efficient measurements from marine vessels
• Land based costal profiling

Furthermore, the ARC-TOP can be used with a winch as a traditional CTD without hardware modifications.

### Hull

The ARC-TOP profiler is divided into three main hull sections as shown in Fig. 1:

• Top hull
• Pressure hull
• Bottom hull

The top and bottom hull are 3D printed by consumer FDM printers in PETG plastic. We have chosen the bright yellow colour for visibility. Ø3 mm carbon fibre rods are bonded to the 3D printed parts, providing strength and mechanical interfaces between the hulls. The pressure hull is machined from EN AW-6082 aluminium.

#### Top hull

The top hull serves as a mounting point for antennas. These are mounted on the very top of the profiler to ensure optimal signal strength upon resurfacing. The top hull also houses the buoyancy foam in an adjustable compartment to adjust the descent velocity.

The pick-up system is also housed in the top hull along with its release mechanism.

#### Pressure hull

The aluminium pressure hull houses all instrument electronics excluding sensors and antennas and provides waterproof interfaces for external components. It is designed for dives to a depth of 400 m at a safety factor of 1.3 determined using finite element analysis. Mechanical testing of the pressure hull and sensors have been limited to 250 m.

**Fig. 4:**
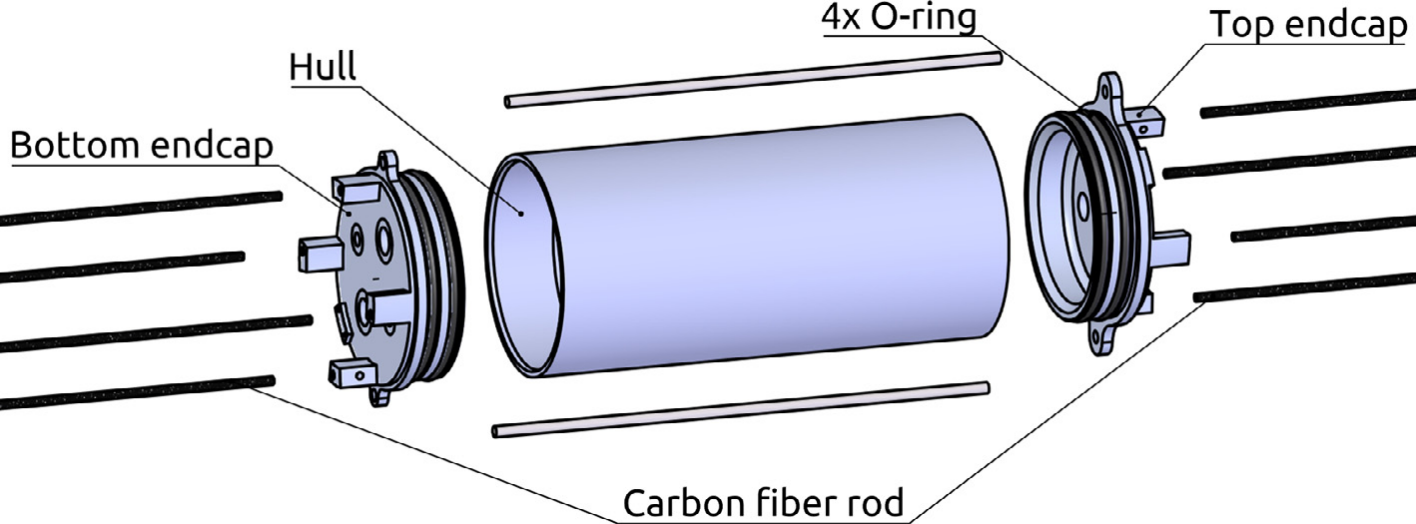
Exploded view of the structural components of the pressure hull.

Three parts make up the pressure hull: Top endcap, cylindrical hull and bottom endcap. We use a double O-ring seal between endcaps and the cylindrical hull. The endcaps have mounting points for the Ø3 mm carbon fibre rods from the top and bottom hulls. A setscrew is used to secure the rods. See Fig. 4.

All three parts of the pressure hull are anodized with a surface thickness of 25 µm. We use a zinc anode on each endcap to provide further protection. Prior to mounting the zinc anode, the anodized surface of contact was sanded down to improve electrical contact between endcap and anode.

Holes in the endcaps provide interfaces for cable penetrators and other equipment. Both Blue Robotics Potted Cable Penetrator [4] and Blue Trail Engineering Simple Penetrator [5] is installed on the instrument. The Potted Cable Penetrator have been potted with WEST SYSTEM G/Flex 650 Toughened Epoxy [6]. Like the hull, these cable penetrators have been tested to a depth of 250 m.

#### Bottom hull

The CTD sensors are housed in the bottom hull, with conductivity and temperature sensors protruding out of the hull for adequate flow speed over the sensing elements. Ballast is mounted to the exterior of the bottom hull, with the release mechanism.

A flexible nose is connected to the bottom hull to provide impact resistance as well as durability during handling the instrument.

#### Stability

**Fig. 5:**
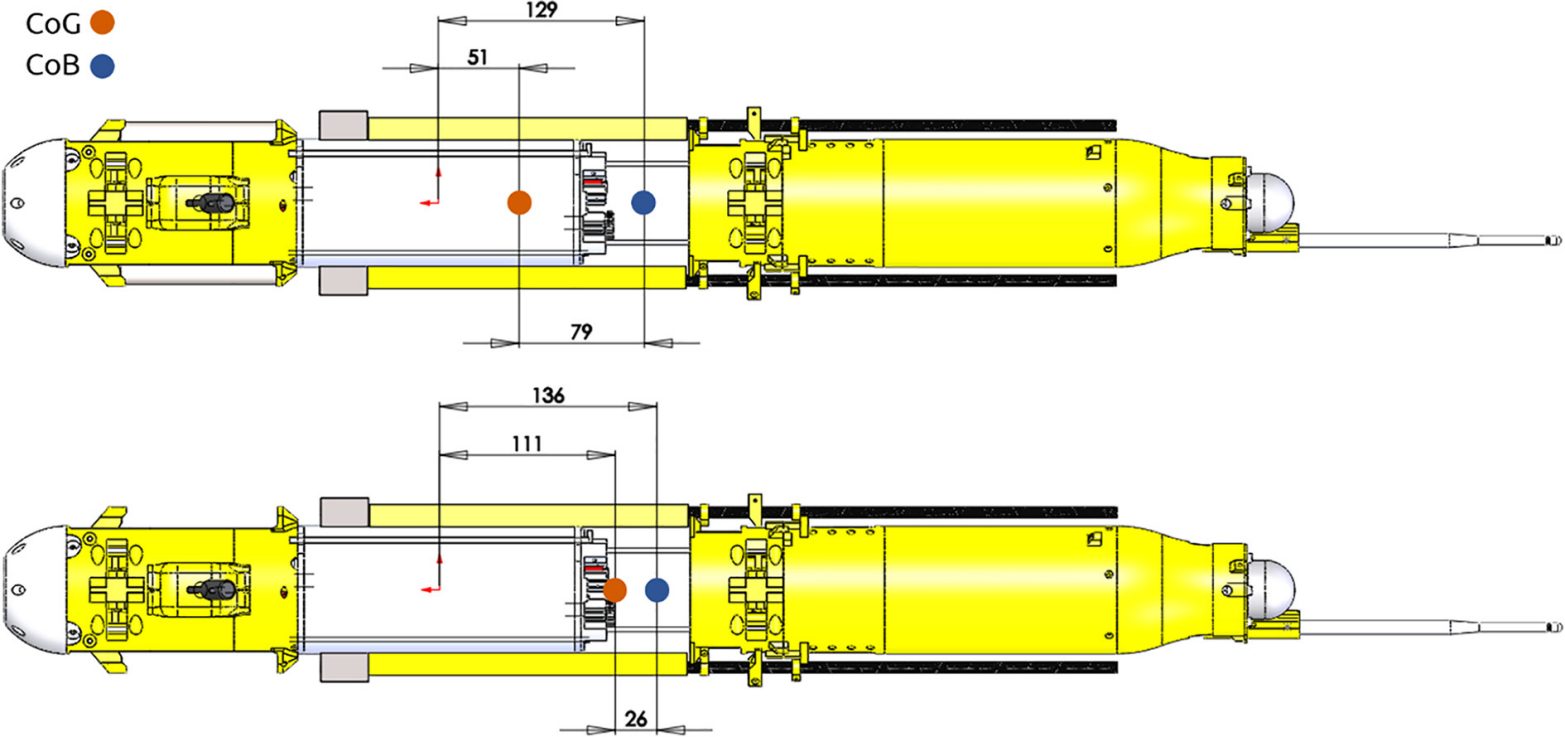
Separation of Center of Gravity and Center of Buoyancy during dive (top) and ascent (bottom). Dimensions in mm.

The layout of the hulls has been designed with consideration of the instrument stability during profiling. This is achieved by having a spatial separation between the Centre of Buoyancy (CoB) and Centre of Gravity (CoG) during both descent (with ballast) and ascent (without ballast). See Fig. 5.

### Buoyancy foam

We use closed cell polyurethane foam to achieve net positive buoyancy without ballast installed [7]. When coated with a waterproof coating (epoxy, latex) this foam provides buoyancy at depths up to 488 m [8].

Fine-tuning the buoyancy of the instrument (i.e. setting the descent/ascent velocity) is possible by using foam discs of varying thickness. The discs are easy to swap out for other thicknesses, and a variable-size compartment have been incorporated into the top hull.

### Buoyancy engine

Changing buoyancy is accomplished by releasing a set of ballast weights. Each ballast weight consists of an Ø20 mm steel rod with a weight in water of 0.2 kg, totalling a change in weight of 0.4 kg at release. When installing the ballast weights, two foam blocks are compressed to provide the releasing force. The actuator provides the retention force until release. With the imposed weight restrictions on the instrument, a very lightweight and simple actuator has been developed. The actuator is designed to be submerged with no sealants and lubrication.

**Fig. 6:**
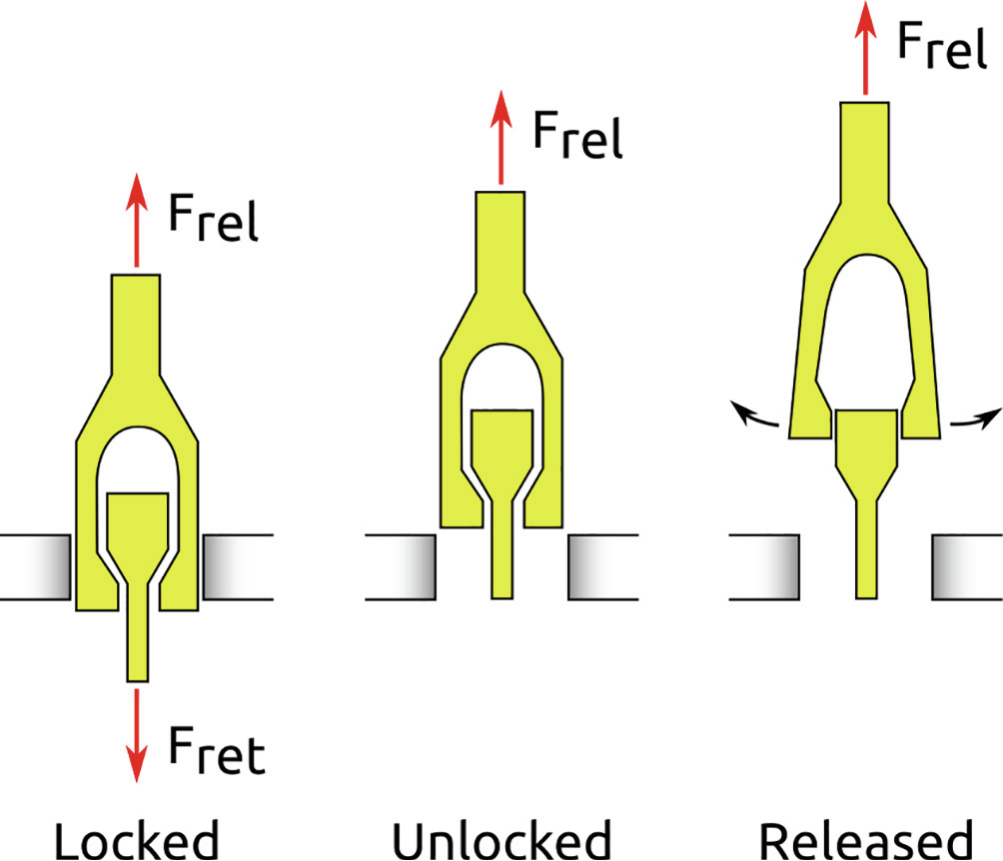
Actuator principle. The retention force (F_ret_) locks the fork between the walls (grey). With the elimination of the retention force, the releasing force (F_rel_) pulls the fork out of the walls and releases it.

**Fig. 7:**
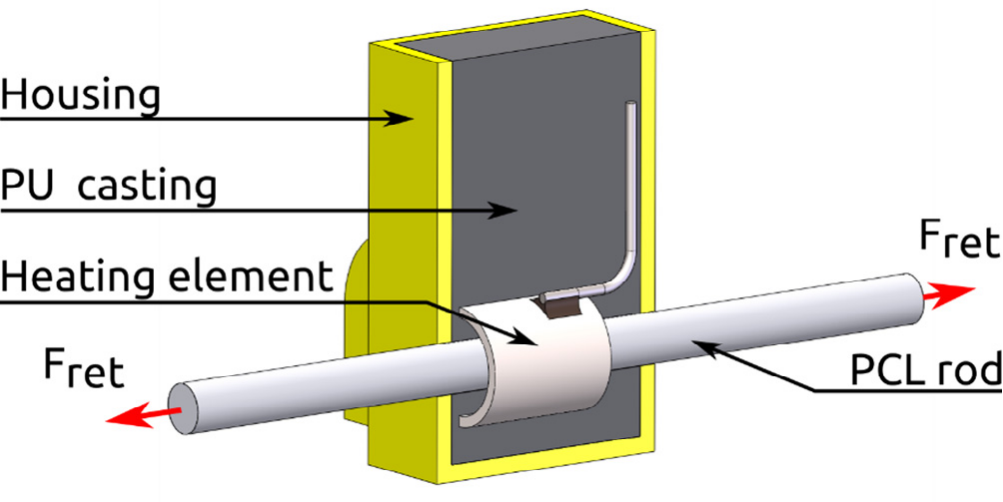
Section view of complete heating element with PCL rod inserted.

A locking mechanism provides the retention force until the actuator introduces a small amount of slack. See Fig. 6. A cylindrical heating element cast in a Polyurethane casing accomplishes actuation by heating an Ø2.8 mm Polycaprolactone (PCL) rod with a melting temperature of approximately 60 °C. The PCL rod elongates when heated and the locking mechanism is unlocked. A section view of the heating element is shown in Fig. 7.

The heating element has a Temperature Coefficient of Resistance (TCR) of 1000 ± 150 ppm/°C. This enables deriving the heating element temperature, T, during use by measuring current and applied voltage (i.e. resistance, R=U/I) by eq. (1):

(1) $$T=\frac{R-R_{0}}{\mathrm{TCR}(R_{0})}+T_{0}$$

where $T_{0}$ and $R_{0}$, are temperature and resistance at calibration, respectively.

The known temperature is used to throttle the heating element by means of PWM signals according to a PID controller to reach a set temperature.

### Pick-up

**Fig. 8:**
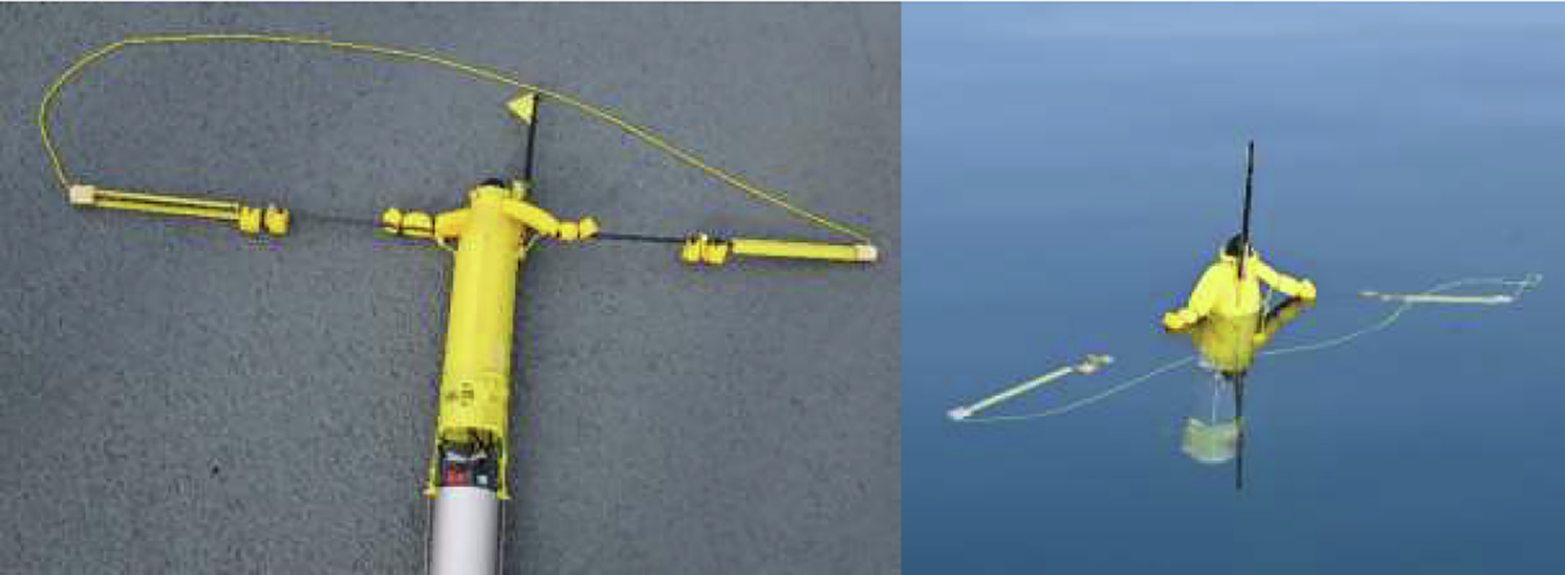
Pick-up arms and wire system for instrument retrieval (left) and wire system position in water (right). The yellow wire is attached to the pressure hull and forms a closed loop that runs through the pick-up arms. Photo courtesy of Peter Bondo Christensen. (For interpretation of the references to colour in this figure legend, the reader is referred to the web version of this article.)

The pick-up system consists of two positive buoyant arms with a closed loop paracord connected to the pressure hull suspended between the arms, as seen in Fig. 8. When ready for deployment, the arms and paracord are fixed to the outside of the top hull by a release mechanism identical to the one used in the buoyancy engine. When deployed the arms float to the surface and extend the paracord between them. A foam block provides durable separation of the arms when extended. This creates a loop that can be caught by a hook mounted to a UAV.

A small wire coupler provides the necessary hardware to mount the ARC-TOP to a winch for non-remote measurement.

### Power

**Fig. 9:**
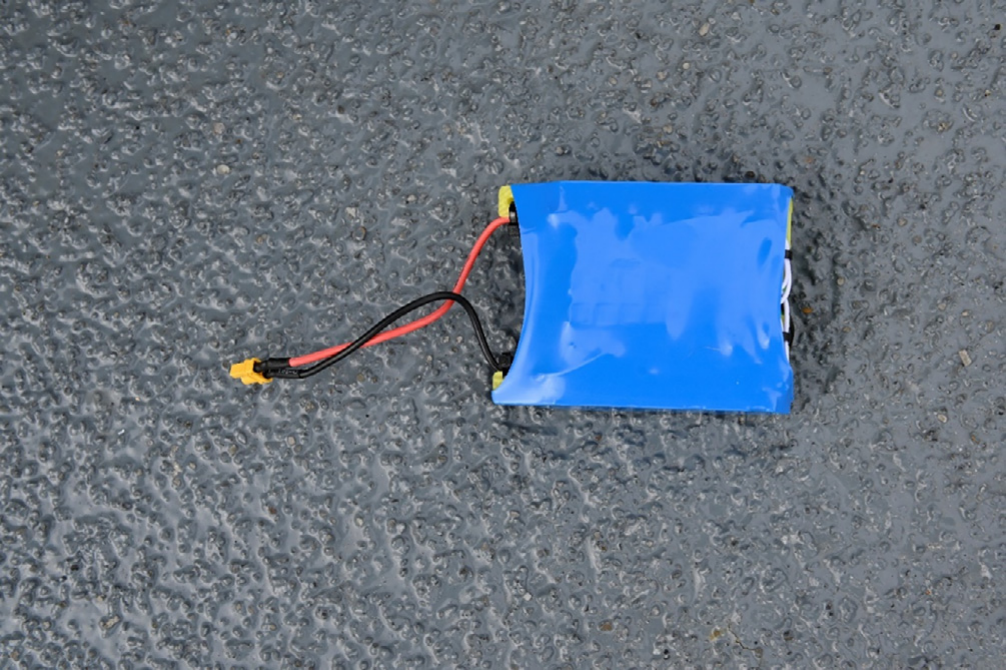
The complete battery pack covered in heat shrink tubing. Photo courtesy of Peter Bondo Christensen.

The ARC-COP is powered by a single battery pack of four 18650 LiPo battery cells [9] in series, resulting in a final rating of 14.8 V and 3000 mAh with 30 A XT30 power connectors. The complete battery pack can be seen in Fig. 9. The cells are spot-welded together and soldered to a common port 20 A Li-ion battery management system (BMS) for cell balancing and protection. To create a rugged package a 3d-printed housing is incorporated into the pack. To reduce the risk of accidental shorts of the battery pack, the entire pack is covered using heat-shrink tubing.

### Sensors and hardware

The essential sensors (conductivity, temperature and pressure) are listed in Table 2. These have been chosen primarily due to their low cost. The CTD sensors sample at a frequency of 1.7 Hz. This is limited only by the Atlas conductivity sensor.

**Table 2:** Main sensor description.

Sensor	Description	Rated accuracy
Temperature	The Blue Robotics Celsius [10] sensor is used to measure temperature in the range −5 °C to 50 °C. The sensing element is incorporated into a convenient housing requiring no user potting.	± 0.1 °C
Pressure	The Blue Robotics Bar30 [11] sensor is used to measure pressure to calculate the instrument depth in water. The sensor range is 0 to 30 bar. The sensing element is incorporated into a convenient housing requiring no user potting.	± 200 mbar
Conductivity	The Atlas Scientific Mini Conductivity Probe K 1.0 and EZO Conductivity Circuit [12], [13] is used to measure conductivity to calculate salinity. The probe range is 5–200,000 μS/cm.	± 1 %

**Fig. 10:**
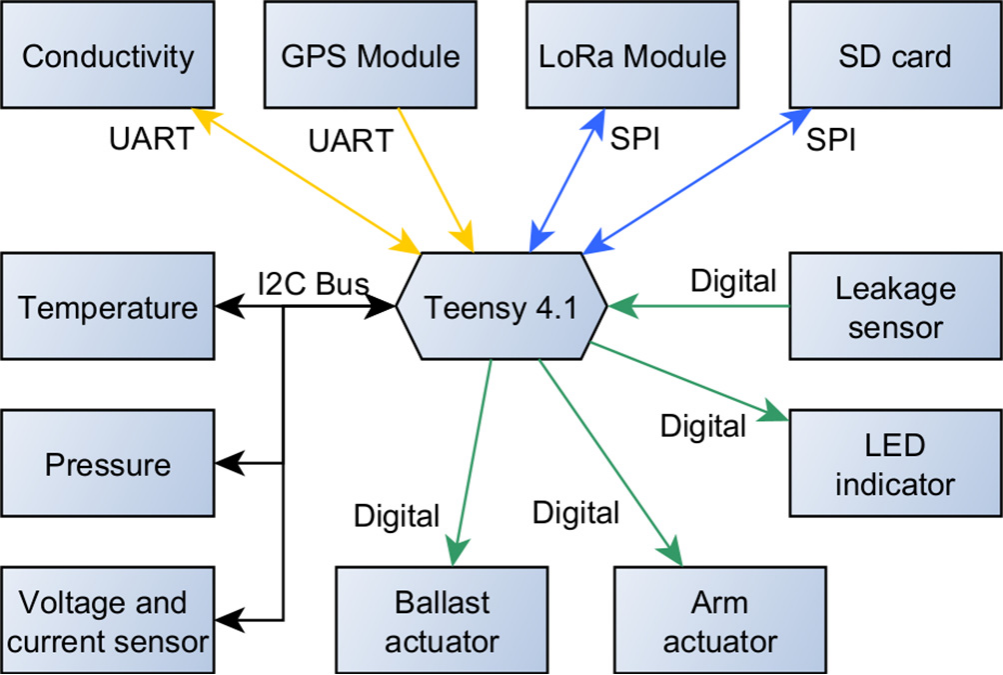
Component overview with communication protocol for the ARC-TOP.

Table 3 provides a description of additional hardware components. Fig. 10 shows all major components and their individual relationships.

**Table 3:** Hardware description.

Hardware	Description
CPU	The Teensy 4.1 development board [14] constitutes the processing unit of the ARC-TOP. This board is compatible with the Arduino software ecosystem [15], enabling rapid development. It has increased processing power, a larger number of interfaces for peripheral devices and a very compact form-factor compared to common Arduino models as well as an on-board Real Time Clock (RTC).
Telemetry	Telemetry between instrument and ground station uses RFM98W 433 MHz transceiver modules [16] that uses the LoRa modulation technique [17] for low power long range communication.
Data storage	A SD card is used along with the built-in Micro SD Socket on the Teensy 4.1 as data logging with the RTC providing timestamps. System debug and event information are also stored here for later review.
Indicator	A Blue Robotics Subsea LED Indicator [18] functions as status indication on the top endcap.
Voltage and current sensor	The Adafruit INA260 voltage and current sensor [19] is used to monitor battery voltage and current applied to the heating elements, as described in section *Buoyancy engine*.
Leakage sensor	The Blue Robotics SOS Leak Sensor [20] and SOS Probes [21] are installed on the ARC-TOP. This is used to measure possible leakage inside the pressure hull at four different locations at the bottom endcap.
GPS	Position measurements are obtained using a Quectel L86-M33 GPS module [22] mounted at the very top to the ARC-TOP in a separate pressure hull. This only has connection when the instrument is at the surface.

### Wiring and PCB

**Fig. 11:**
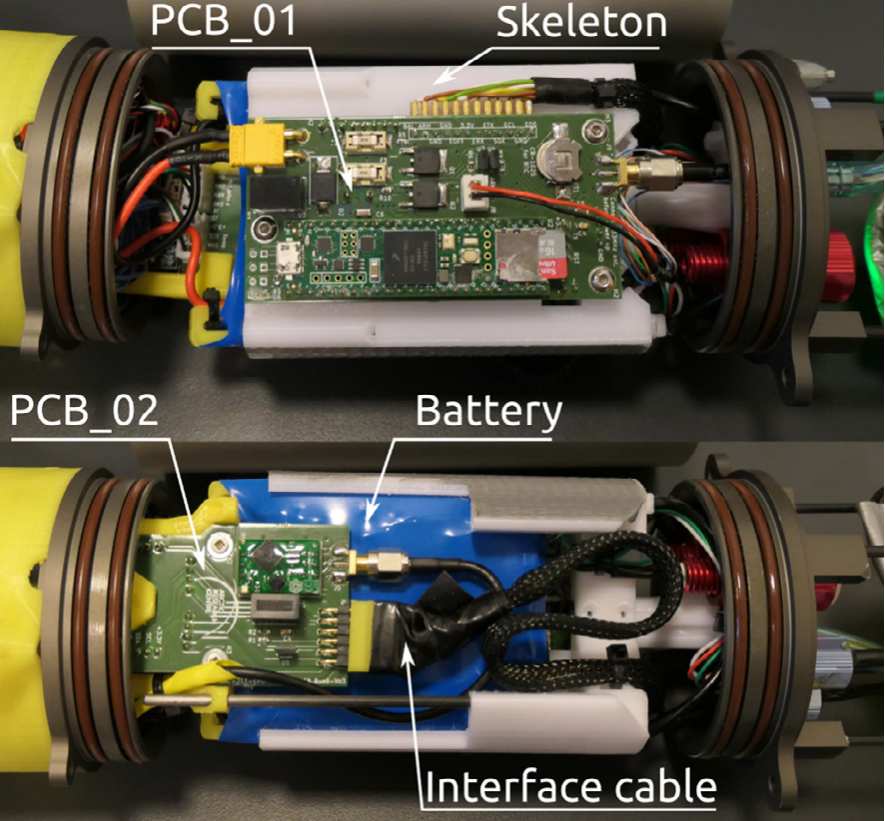
PCBs, wiring and skeleton of the pressure hull seen from the front and back with hull removed.

Spatial requirements within the pressure hull dictates a very compact electric design with strict wire planning. Wires enter the pressure hull from both endcaps, and this presents a design challenge concerning easy disassembly and assembly. The resulting design uses two 2-layer printed circuit boards (PCBs) connected to each their own endcap with a single 12-wire cable as interface to alleviate these requirements. Fig. 11 shows the pressure hull PCBs, internal skeleton, and interface cable between PCBs.

**Fig. 12:**
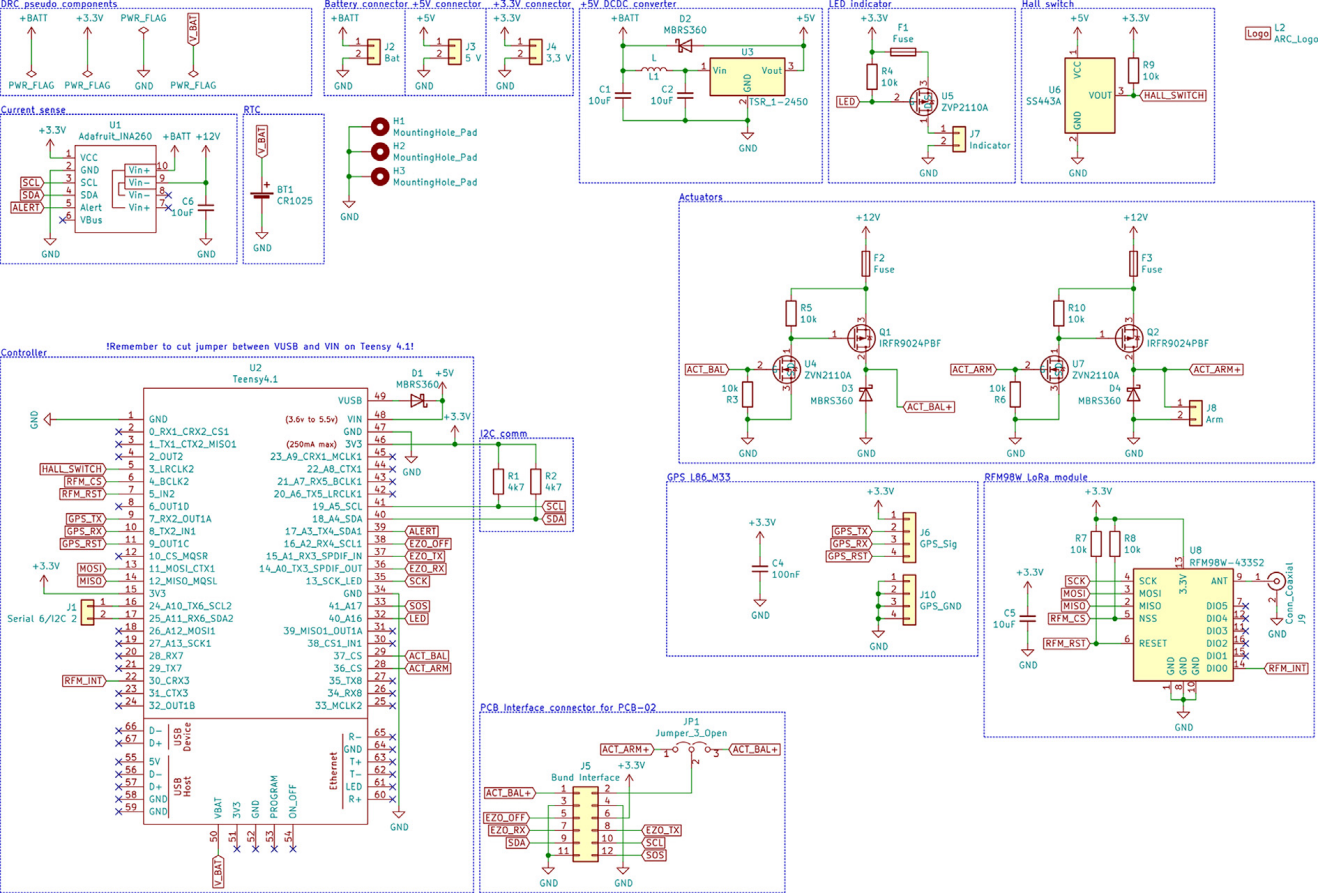
Schematic for PCB_01.

**Fig. 13:**
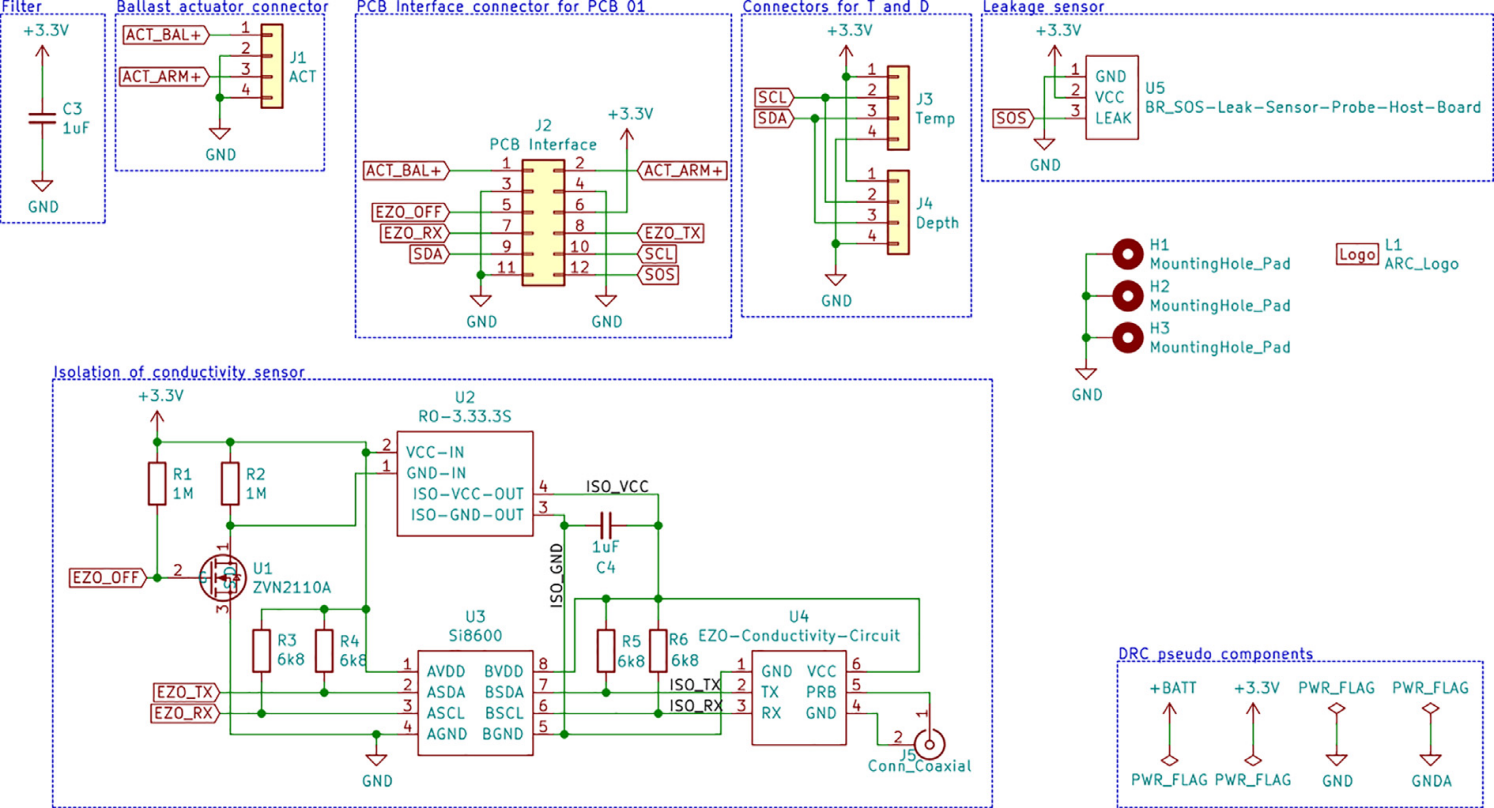
Schematic for PCB_02.

PCB_01 houses the Teensy microcontroller board and control electronics. PCB_02 is mainly used for connectors and electrical isolation for the conductivity sensor. Schematics for both PCBs can be seen in Fig. 12 and Fig. 13.

### UAV

Custom mounting hardware and release mechanisms for the ARC-TOP have been designed for the UAV used. Modifying the mounting hardware for other UAVs is possible by simply exchanging a few 3d printed parts to fit the specific use-case. The electric design allows connecting to different UAV autopilot units with little to no configuration.

The ARC-TOP is mounted below the UAV with a strap that can be released by servo actuation.

A DJI Digital FPV System camera and transmitter [23] is mounted at the front of the mounting hardware to enable remote operation. A hook is mounted at the rear for retrieval of the instrument after profiling. Both are operated by servos.

A MaxBotix MB1242 ultrasonic rangefinder [24] is used to overlay aircraft altitude on the pilot display for accurate instrument drop height.

### Software

All software is written in the Arduino programming language. The software is designed as a state-machine that, when armed, autonomously navigates the appropriate states to complete a profile. Table 4 describes each state along with the conditions for entering the state.

**Table 4:** Software states ordered by normal operating flow.

State	Description	Entered if
Start-up	Initialize peripherals and communications, check for errors, report status and enter Idle or Error state based on status	Power on
Idle	Instrument is ready to accept commands, while continuously monitoring position and system status	Either: Successful start-up Disarming profiler Reload ballast/arm complete
Reload ballast/arm	Reload actuator by melting used PCL material for easy removal. This is done by heating the heating element to a predefined temperature using a PID controller.	Received reload ballast/arm command and system state is idle
Armed	Ready the instrument for drop and flight by UAV: Read CTD sensors Update position based on GPS input Broadcast instrument status by LoRa Check for incoming commands Enter Dive state if dropped from UAV	State is idle and received arm command
Dive	Measures CTD downcast profile and continuously monitors if the dive should be aborted by setting Release ballast state if: Depth reached Leak detected Max dive time elapsed	Detected drop from UAV in arm state
Release ballast	Release ballast to start ascent by heating the heating element. Repeat if depth is not decreasing. Set ascent state when complete	Dive completed/aborted
Ascent	Measures CTD upcast profile and check if the instrument has reached the surface: Set state Release arm if surface reached Set state Low power surface if max ascent time elapsed	Decreasing depth detected after releasing ballast
Release arm	Release arms to enable pickup by heating the heating element. Sets state Surface when complete	Surface detected after ascent
Low power surface	Wait for reaching surface with non-essential systems shut down for low power consumption. Set state Release arm if surface reached	Surface not detected after ascent
Surface	Update position based on GPS input and broadcast it using LoRa radio along with system status	Release arm complete
Error	Broadcast error message while monitoring system status	Error detected

The status indicator LED is cycled at a frequency and duty cycle unique to the individual software states using interrupts at all times when the instrument is turned on.

#### Data logging

All data is saved to the SD card in a single CSV file per dive for later download. Logging information is stored in a single text file per dive.

Depth measurements are logged in meters, temperature in degrees Celsius and salinity in PSU.

### Price

**Fig. 14:**
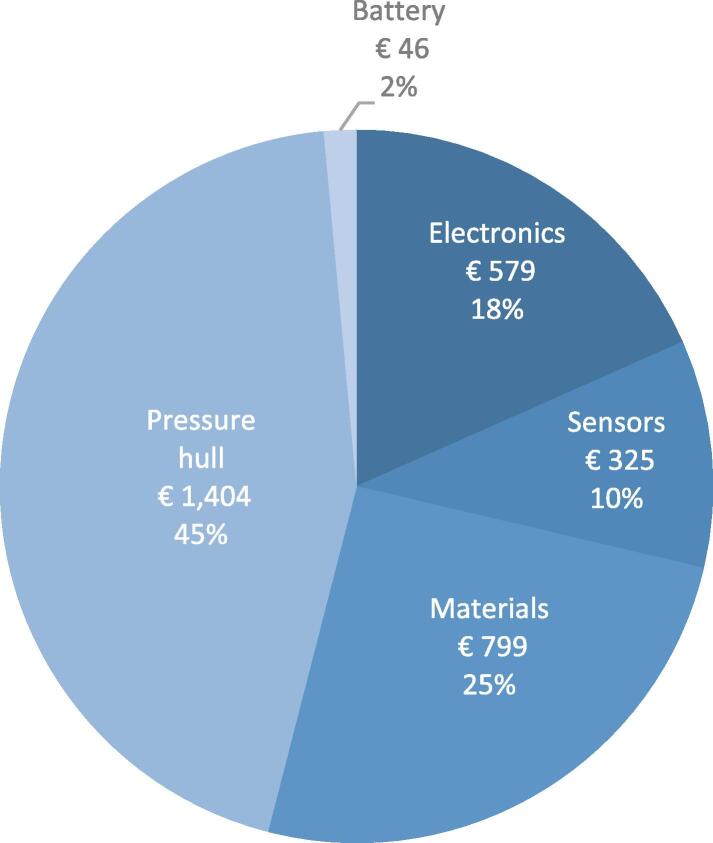
Price overview of the ARC-TOP ex. VAT.

Using consumer grade components and sensors and 3D-printed hull sections keeps the price of the ARC-TOP at a minimum, totalling € 3153. Low price is essential for use in high-risk areas. An overview of system cost is seen in Fig. 14. The listed cost is based on the production of two sensor units and a single UAV mounting hardware unit. Cost of development and assembly is not included.

The pressure hull accounts for 45 % of the total cost, due to the custom machined components. At larger production volume the cost of custom parts decreases greatly. Exact figures for various production volumes have not been calculated. Cost of individual components can be found in the attached BOM. In-house production cost has been estimated on an hourly wage of € 67.

## Design files summary

Design files for construction and further development have been uploaded to an online repository (https://doi.org/10.17632/zdvb5hzv2x.1). Each folder in the repository contains a read-me file with information specific to the design files in this folder.

A summary of the main design files are listed in Table 5. A complete list is found in the file *Naming_and_BOM.xlsx*.

**Table 5:** Design file summary. Listed files are the recommended files for design file overview. Other design files are linked inside the listed files.

Design file name	File type	Open source license	Location of the file
Naming_and_BOM.xlsx	Excel Workbook	CC BY 4.0	https://doi.org/10.17632/zdvb5hzv2x.1
TOP-200-Model.step	CAD file	CC BY 4.0	https://doi.org/10.17632/zdvb5hzv2x.1
TOP-300-Model.step	CAD file	CC BY 4.0	https://doi.org/10.17632/zdvb5hzv2x.1
TOP-220-PCB_01.pro	KiCad Project	CC BY 4.0	https://doi.org/10.17632/zdvb5hzv2x.1
TOP-220-PCB_02.pro	KiCad Project	CC BY 4.0	https://doi.org/10.17632/zdvb5hzv2x.1
TOP-320-PCB_03.pro	KiCad Project	CC BY 4.0	https://doi.org/10.17632/zdvb5hzv2x.1
TOP-420-PCB_04.pro	KiCad Project	CC BY 4.0	https://doi.org/10.17632/zdvb5hzv2x.1
KiCad library	Directory	CC BY 4.0	https://doi.org/10.17632/zdvb5hzv2x.1
Solution.sln	Visual Studio solution	CC BY 4.0	https://doi.org/10.17632/zdvb5hzv2x.1
TOP-220-Controller.ino	Arduino Software file	CC BY 4.0	https://doi.org/10.17632/zdvb5hzv2x.1
TOP-320-Controller.ino	Arduino Software file	CC BY 4.0	https://doi.org/10.17632/zdvb5hzv2x.1
TOP-420-Controller.ino	Arduino Software file	CC BY 4.0	https://doi.org/10.17632/zdvb5hzv2x.1

The *Naming_and_BOM.xlsx* Excel Workbook contains helpful information regarding the design files by supplying a naming convention used for all files and a complete list of all relevant files. Bill of Material information is also listed here.

*TOP-200-Model.step* is a complete CAD file for all components in the ARC-TOP profiler. A SolidWorks assembly is also provided in the same location.

*TOP-300-Model.step* is a complete CAD file for all components in the ARC-TOP UAV mounting hardware. A SolidWorks assembly is also provided in the same location.

*TOP-220-PCB_01.pro* through *TOP-420-PCB_04.pro* are KiCad project files for PCB manufacturing of all circuit boards for the entire solution (profiler, UAV, ground station). Relevant library components (schematics and footprints) are located in the *KiCad library* directory.

The free code editor Visual Studio Community [25] have been used extensively throughout software development, along with the plugin for Arduino development Visual Micro [26]. Visual Studio organizes code in “Solutions” that contain all necessary links between projects and libraries. It is recommended to use the file *Solution.sln* to inspect the code. *TOP-220-Controller.ino*, *TOP-320-Controller.ino* and *TOP-420-Controller.ino* are the projects uploaded to the microcontrollers of the profiler, UAV and ground station, respectively.

## Bill of materials summary

A summary of the main components is listed as a BOM in Table 6. A complete BOM can be found in the root of the supplied repository in the file *Naming_and_BOM.xlsx*.

**Table 6:** Bill of Materials summary. A complete BOM can be found in the supplied repository with the name “Naming_and_BOM.xlsx”.

Designator	Component	Number	Cost per unit - EUR ex. VAT	Total cost - EUR ex. VAT	Source of materials	Material type
P01	Mini Conductivity Probe K 1.0	1	100.65	100.65	https://atlas-scientific.com	Semi-conductor
P02	EZO Conductivity circuit	1	54.73	54.73	https://atlas-scientific.com	Semi-conductor
P03	Potted Cable Penetrator, M6	4	3.53	14.13	https://bluerobotics.com	Metal
P04	West System epoxy G/flex 650–8	1	33.24	33.24	https://www.hfmarine.dk	Polymer
P05	RG174A/U 50 Ohm Coax cable 20 m	1	22.98	22.98	https://dk.rs-online.com	Semi-conductor
P06	24 AWG, 4 conductor PU cable	1	4.41	4.41	https://bluerobotics.com	Metal
P07	Cylindrical ceramic heating element	2	6.24	12.48	Zhuhai 4U Electronic Ceramics Co.	Ceramic
P08	DiaCast 76 PUR Casting rubber	1	20.87	20.87	https://www.diatom.dk	Polymer
P09	SMA Female crimp connector, RG-174	3	3.77	11.30	https://dk.rs-online.com	Metal
P10	433 MHz antenna	3	8.60	25.80	https://www.mouser.dk	Semi-conductor
P11	Quectel GPS receiver	1	16.80	16.80	https://dk.rs-online.com	Semi-conductor
P12	O-ring 33 X 2 NBR	1	0.64	0.64	https://ehandel.mw.dk	Polymer
P13	Simple Penetrator M10	4	15.01	60.04	https://www.bluetrailengineering.com	Metal
P14	TOP-211-Endcap_top	1	389.81	389.81	Nymark & Fogh Teknik	Metal
P15	TOP-211-Endcap_bottom	1	389.81	389.81	Nymark & Fogh Teknik	Metal
P16	Polyurethane Tubing Ø8mm, 1 m	1	3.53	3.53	https://www.bluetrailengineering.com	Polymer
P17	Celsius Fast-Response Temperature Sensor, ±0.1 °C	1	52.98	52.98	https://bluerobotics.com	Semi-conductor
P18	Subsea LED indicator	1	11.48	11.48	https://bluerobotics.com	Semi-conductor
P19	M10 enclosure vent and plug	1	7.95	7.95	https://bluerobotics.com	Metal
P20	Bar30 High-Resolution Pressure Sensor, 300 m	1	63.57	63.57	https://bluerobotics.com	Semi-conductor
P21	VEC™- Carbon fibre rod Ø3mm 1 m	2	1.82	3.63	https://ekomposit.dk	Composite
P22	Closed cell PU foam 20x30x810mm	1	6.72	6.72	Arbi-Skum	Polymer
P23	Teensy 4.1 development board	1	27.57	27.57	https://www.elfadistrelec.dk	Semi-conductor
P24	2.54 mm pitch socket head male header, right angle	2	5.46	10.92	https://www.mouser.dk	Metal
P25	2.54 mm pitch socket head female header, double row	2	55.69	111.38	https://www.digikey.dk	Metal
P26	VEC™- Carbon fibre tube Ø7mm 1 m	2	6.35	12.69	https://ekomposit.dk	Composite
P27	SOS Leak sensor	1	25.61	25.61	https://bluerobotics.com	Semi-conductor
P28	Paracord Ø1.9 mm 1 m	15	0.28	4.20	https://www.paracord.eu	Polymer
P29	18650 lithium battery cell, 3.7 V, 3Ah	4	8.23	32.92	https://www.conradelektronik.dk	Metal
P30	Li-ion 4S 20A Li-ion BMS	1	10.65	10.65	https://www.batteri-energi.dk	Semi-conductor
P31	XT30 connector, female	1	0.70	0.70	https://www.conradelektronik.dk	Metal
P32	Heat shrink tubing, 120 mm	1	2.17	2.17	https://batteri-energi.dk	Polymer
P33	Carbon fibre plate, cured	1	12.48	12.48	https://ekomposit.dk	Composite
P34	Nylon Webbing	1	6.35	6.35	https://www.jemogfix.dk	Polymer
P35	TOP-211-Canister	1	389.81	389.81	Nymark & Fogh Teknik	Metal
P36	Threaded rod, M4	1	6.14	6.14	https://ehandel.mw.dk	Metal

Parts in the BOM that have a listed price of € 0 is due to in-house production (mainly 3D-printing and simple cutting of materials). This is naturally not applicable if an external manufacturing service is used. The cost of materials for these parts is included in the BOM as a separate entry.

## Build instructions

**Fig. 15:**
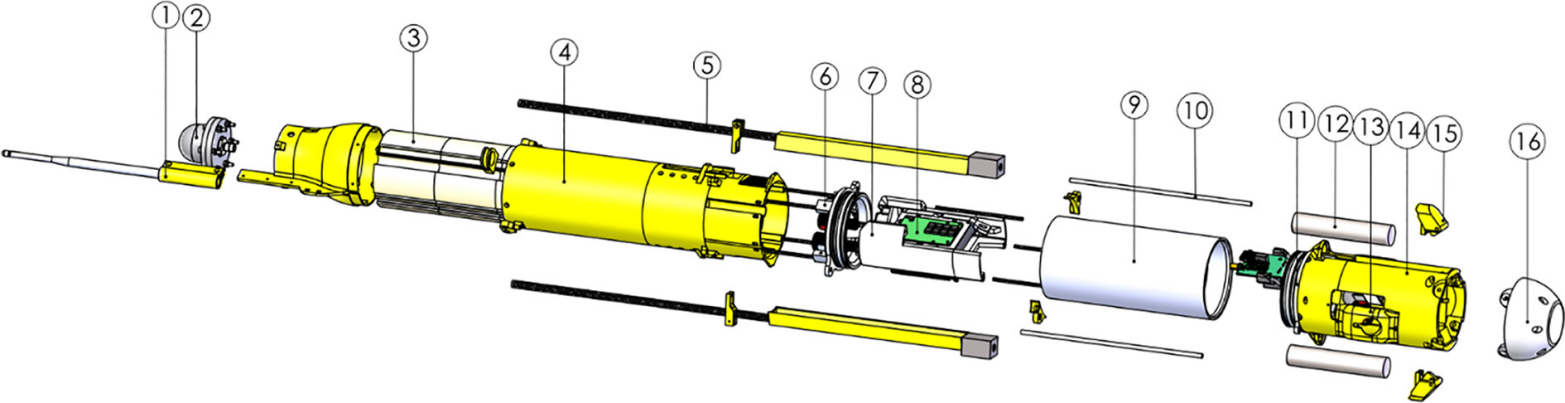
General overview of the ARC-TOP during assembly with marked LoRa antenna (1), GPS antenna (2), buoyancy foam (3), top hull (4), pick-up arms (5), top endcap with penetrators (6), electronics frame (7), battery (8), pressure hull (9), pressure hull fastener (10), bottom endcap with penetrators (11), ballast (12), sensor mount (13), bottom hull (14), ballast cap (15) and nose (16).

The general assembly procedure for the ARC-TOP is to assemble the pressure hull first with all cable penetrators attached. Once complete, the top and bottom hulls should be assembled and mated with the pressure hull. An exploded view of the profiler can be seen in Fig. 15 as a reference during assembly. It is highly recommended to use the provided CAD files for component placement and identification.

Detailed assembly instructions for each subassembly follows below. General guidelines throughout manufacturing:

• Carbon fibre parts will need to be cut and shaped during assembly. Please use appropriate protective equipment.
• Casting and potting using epoxy resin and polyurethanes is required along with alcohol and acetone. These chemicals are hazardous and should be handled using required safety measures.
• Soldering electronics is part of the assembly process. Use a well-ventilated area to prevent build-up of fumes.
• O-rings should be inspected before installing to check for any damage or contaminants. All O-rings should be lubricated using an appropriate lubricant (we use Molykote 111 Compound [27]).
• Any fastener that is in contact with water should be of A4 grade stainless steel.
• When assembling and handling the ARC-TOP take care not to damage any wires from tools or sharp edges. A small cut in the insulation can cause a leak.
• The PU tube used with the Simple Penetrator can be disassembled for fine tuning tubing length or maintenance by cutting the tubing around the spigot with a wire cutter. Caution must be exercised not to damage the Simple Penetrator as this can impede the performance of the penetrator. Refer to the manufacturer guidelines [28].

Technical drawings are available in the supplied repository for machined parts. Screws, bolts and nuts used during assembly are listed in Table 7.

**Table 7:** List of fasteners used during assembly.

Standard	Size	Description	Purpose
DIN 7982	2.2X6.5	Countersunk self-tapping screw	Plastic
DIN 7982	2.2X9.5	Countersunk self-tapping screw	Plastic
DIN 7982	4.2X9.5	Countersunk self-tapping screw	Plastic
DIN 912	3X35	Cylindrical socket head screw	Bolted joints
DIN 912	3X20	Cylindrical socket head screw	Bolted joints
DIN 985	M3	Locking nut	Fastening
DIN 985	M4	Locking nut	Fastening
DIN 975	M4	Threaded rod	Pressure hull securing

**Table 8:** Cable lengths of potted penetrators. External lengths is the length from external cable end to external face of the penetrator (cup end). Internal lengths is the length from internal cable end to internal face of the penetrator (threaded end).

Component	Wire	External length [mm]	Internal length [mm]	Total length [mm]
LoRa	Coaxial (P05)	500	300	800
Actuator, top	PU Cable (P06)	320	250	570
Actuator, bottom	PU Cable (P06)	320	180	500
Mini Conductivity Probe K 1.0 (P01)	Coaxial, supplied with probe	260	Rest	–

### Aluminium parts

The following parts should be hard anodized to a thickness of min. 25 μm to prevent accidental damage to the protective layer during use:

- TOP-211-Canister
- TOP-211-Endcap_bottom
- TOP-211-Endcap_top
- TOP-310-Hook_arm
- TOP-310-Hook_tube

See supplied technical drawings for manufacturing instructions.

### 3D-printed parts

The supplied BOM contains list of all 3D-printed parts and their quantity. These have been printed on consumer grade FDM printers. The used material is also listed. After printing any support material must be removed and print defects should be corrected if possible.

### Potting

Prior to potting, make sure the Atlas Mini Conductivity Probe K 1.0 and EZO Conductivity circuit (P01-2) return reliable readings. Continue with potting:

1. Cut the Mini Conductivity Probe K 1.0 connector off to allow insertion into the penetrator.
2. Cut wire to length according to Table 8. The PU insulation of the PU Cable must be removed on the internal part.
3. Lightly abrade the wire with 80-grit sandpaper and wipe with acetone at the location of the penetrator (according to Table 8) to improve bonding.
4. Clean the penetrator (P03) potting face with rubbing alcohol. Avoid touching this face afterwards to prevent contaminating the bonding.
5. Thoroughly abrade the potting face of the penetrator with 80-grit sandpaper immediately prior to potting. Continue until through the blue anodizing to improve bond strength.
6. Clean the abraded surface multiple times with acetone.
7. Insert wire into penetrator with the abraded part of the wire aligning with the penetrator and mount in a fixture that holds the penetrator upright while fixing the wire.
8. If necessary, use tacky tape to seal the internal part of the penetrator to prevent epoxy running through.
9. Mix G/flex epoxy (P04) in the ratio specified by the manufacturer. This epoxy has a high viscosity and extra care must be taken no to introduce air into the epoxy when mixing.
10. Pour epoxy into the penetrator. Be careful not to introduce air into the epoxy. Lightly tap the fixture or the penetrators to help the epoxy flow into the penetrator and to help air escape.
11. Wait for the epoxy to fully cure before removing from the fixture.

**Fig. 16:**
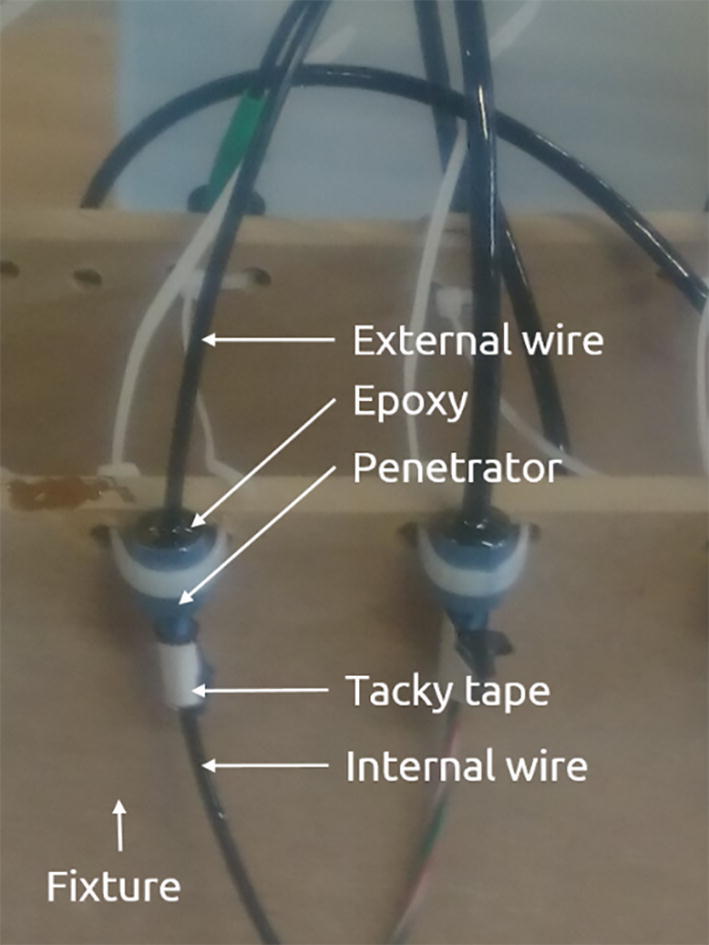
Completed potting of penetrator.

The complete potting of two penetrators can be seen in Fig. 16.

### Heating elements

1. Print the enclosure *TOP-250-Heating_element_casting* and clean the internal compartment from any strings produced during printing.
2. Strip back 5 mm of the external wire prepared during potting above and solder the heating element (P07) to the wire. Use heat shrink tubing to prevent shorting.
3. Lightly abrade the wire near the heating element with 80-grit sandpaper and wipe with acetone to improve bonding to the PUR casting rubber (P08).
4. Insert and fix the heating element inside the printed enclosure with the cylindrical heating element being concentric with the holes in the enclosure. Make sure the heating element is not in contact with the enclosure.
5. Insert a smooth Ø3 mm rod into the holes in the enclosure and through the heating element. We used a M3 bolt with a long non-threaded shaft.
6. Mix the PUR casting rubber in the ratio specified by the manufacturer. Mix slowly to prevent introducing air into the mixture.
7. Pour mixture into the enclosure and wait for it to fully harden before handling.
8. Carefully remove the Ø3 mm rod from the casting by twisting while pulling.
9. Cut off the bottom stabilizing surface and the wire fix tab.

### LoRa antenna

1. Print the enclosure *TOP-241-LoRa_antenna_housing* and clean the internal compartment from any strings produced during printing.
2. Install a female SMA (P09) connector on the external coaxial wire prepared during potting above and connect the wire to the 433 MHz antenna (P10).
3. Lightly abrade the wire near the heating element with 80-grit sandpaper and wipe both the wire and the antenna with acetone to improve bonding to the PUR casting rubber.
4. Insert the antenna and wire through the bottom of the printed enclosure and seal the bottom with tacky tape. Make sure the antenna does not touch the sides of the enclosure.
5. Mix the PUR casting rubber in the ratio specified by the manufacturer. Mix slowly to prevent introducing air into the mixture.
6. Pour mixture into the top of the enclosure and wait for it to fully harden before handling.
7. Cut off the bottom stabilizing surface.

### Anode

Two zinc anodes were made in-house using the dimensions of the part *TOP-211-Anode*. Tolerances are generally very high.

### GPS canister

**Fig. 17:**
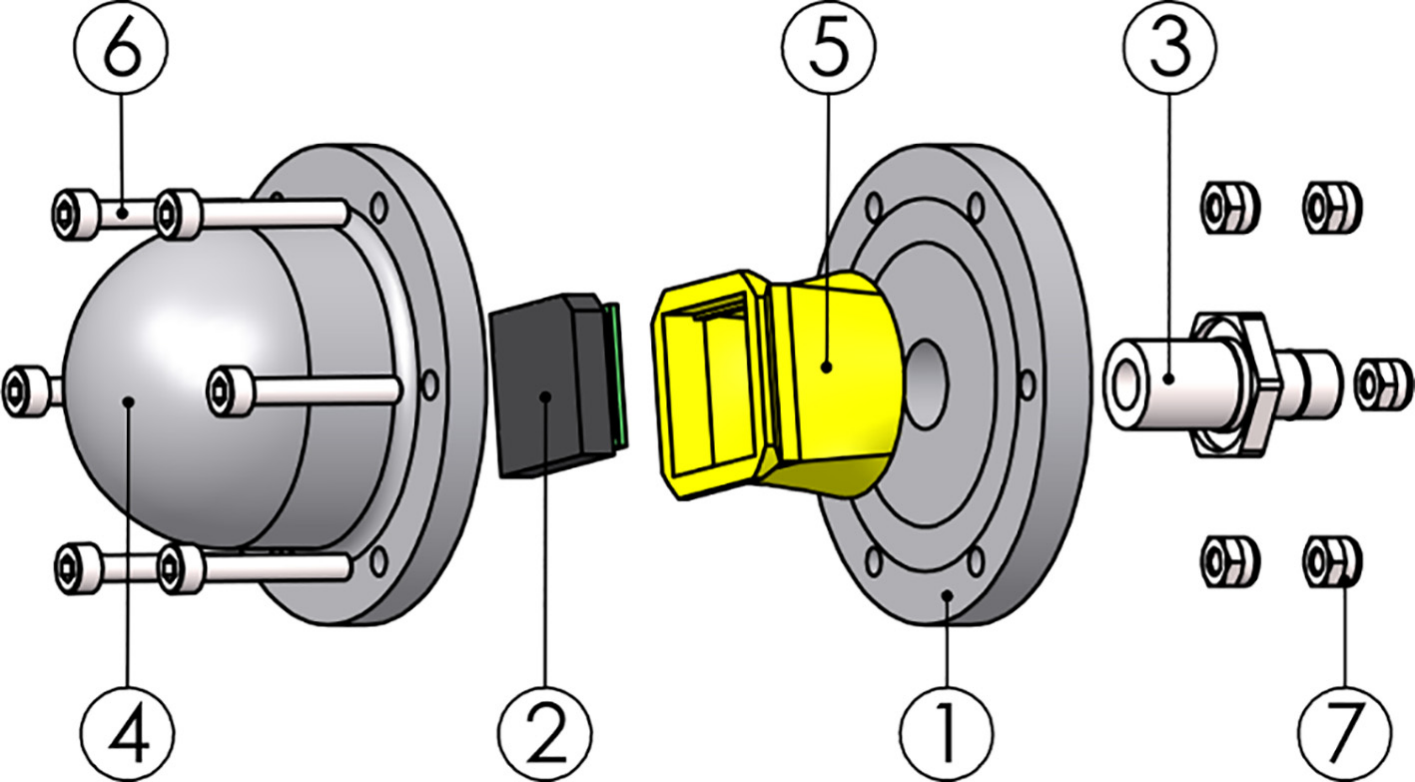
Exploded view of the GPS hull. Parts are: TOP-233-GPS_antenna_hull_endcap (1), GPS module (P11) (2), Simple Penetrator (P13) (3), TOP-233-GPS_antenna_hull (4), TOP-233-GPS_antenna_mount (5), M3x20 A4 bolt (6), M3 A4 locking nut (7).

Use the exploded view in Fig. 17 as a reference during assembly.

1. Solder twisted pair wires with ground on all pairs to the GPS module (P11) pins RXD1, TXD1, Reset and VCC. See the module datasheet [29]. We used Cat 5e network cable as this is supplied as twisted pairs.
2. Solder the ground wires from the pairs to the GND pins.
3. Short the V_BCKP pin to VCC on the module.
4. Use glue as stress relief for the wires, bending the wires towards the centre of the module.
5. Assemble according to Fig. 17. Remember to install the O-ring (P12) and nut for the Simple Penetrator (P13) during assembly (not shown).

### Pressure hull

1. Sand down the anode mounting points on both endcaps (P14-15) to remove the anodizing to improve electrical connection between hull and anode.
2. Clean the mounting surfaces and mount the anodes.
3. Check the electrical connections using an Ohmmeter.
4. On the top endcap (P14):
  a. Mount the penetrators to the endcap using Fig. 18 as reference.
    i. Install part *TOP-211-Electronics_frame_top* before installing the nuts on the inside face of the endcap.

**Fig. 18:**
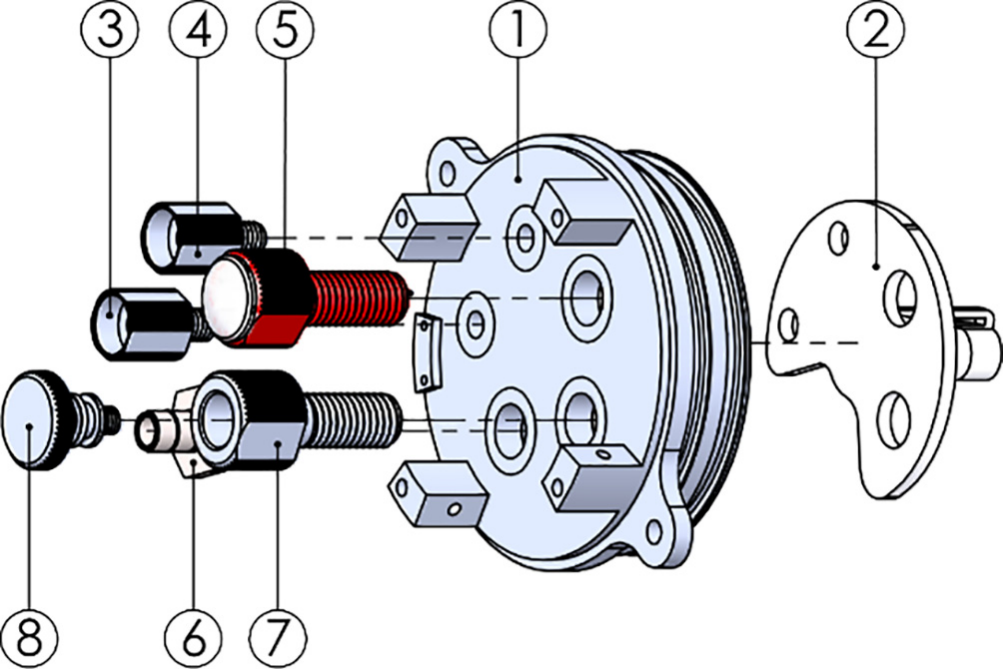
Top endcap assembly with part TOP-211-Endcap_top (1), TOP-211-Electronics_frame_top (2), potted LoRa antenna (3), potted heating element (4), indicator (P18) (5), Simple Penetrator (P13) (6) and Vent Plug (P19) (7–8).
  b. Remember to insert the O-rings supplied with the penetrators.
  c. Install PU tube (P16) of approx. 70 cm on the Simple Penetrator
  d. Thread the wires from the GPS assembled above into the open end of the PU tube, without inserting the GPS-end Simple Penetrator into the tube.
5. On the bottom endcap (P15):
  a. Mount the penetrators to the endcap using Fig. 19 as reference.
    i. Install part *TOP-211-Electronics_frame_bottom* before installing the nuts on the inside face of the endcap.

**Fig. 19:**
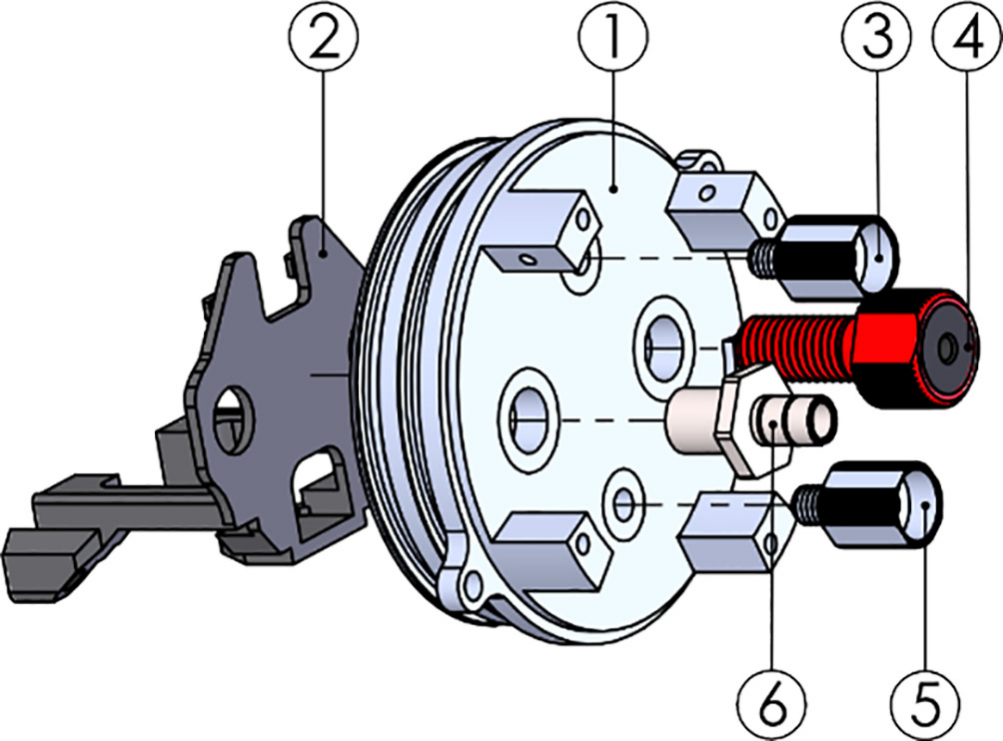
Bottom endcap assembly with part TOP-211-Endcap_bottom (1), TOP-211-Electronics_frame_bottom (2), potted Mini Conductivity Probe K 1.0 (3), Bar30 pressure sensor (P20) (4), potted heating element (5), and Simple Penetrator (P13) (6).
  b. Remember to insert the O-rings supplied with the penetrators.
  c. Install PU tube of approx. 10 cm on the Simple Penetrator
  d. Mount the Celsius temperature sensor (P17) with O-ring in the part *TOP-231-Temp_coupler*. Mount the Simple Penetrator with O-ring to the other end by threading the wire through the penetrator.
  e. Thread the wires from the temperature assembly into the open end of the PU tube, without inserting the temperature-end Simple Penetrator into the tube.
6. Cut the threaded rod (P36) into two pieces of 190 mm.

### Top hull

1. Insert and fasten parts *TOP-250-Lever* and *TOP-250-Locking_arm_top* in *TOP-211-Top_hull_03* with M3 bolts and locking nuts. Do not tighten too much, as arms need to pivot freely.
2. Insert the top heating element in the part *TOP-211-Top_hull_03* using M3 bolts and locking nuts.
3. Route the wires for the GPS and LoRa antennas on the outside *of TOP-211-Top_hull_03* in the slots provided.
4. Cut four Ø3 mm carbon rods (P21) to a length of 324 mm. Glue them into *TOP-211-Top_hull_01* and *TOP-211-Top_hull_02*.
5. Slide the GPS and LoRa antennas through the assembly and push the carbon fibre rods through *TOP-211-Top_hull_03* and into the top endcap (P14). Install and tighten M3 set screws to secure the top hull to the endcap.
6. Install part *TOP-211-Buoyancy_foam_adjuster* into part *TOP-211-Top_hull_02*.
7. Insert buoyancy foam into the top hull with the antenna wires in the designed slots.
8. Before inserting the part *TOP-211-Top_hull_antenna_mount* check the length of the PU tube and shorten if necessary. Be careful not to damage the wires. Insert the GPS-end Simple Penetrator into the PU tube.
9. Route the GPS antenna through the centre of the part *TOP-211-Top_hull_antenna_mount* with the LoRa antenna on the outside in the designated mounting bracket.

### Bottom hull

1. Insert and fasten parts *TOP-250-Lever* and *TOP-250-Locking_arm_bottom* in *TOP-211-Bottom_hull_02* with M3 bolts and locking nuts. Do not tighten too much, as arms need to pivot freely.
2. Insert the bottom heating element using M3 bolts and locking nuts.
3. Cut four Ø3 mm carbon rods to a length of 126 mm. Glue them into *TOP-211-Bottom_hull_01* and *TOP-211-Bottom_hull_02*.
4. Install the bottom hull on the endcap. Install and tighten M3 set screws to secure the hull.
5. Route the Mini Conductivity Probe K 1.0 and Celsius temperature sensor out the holes in the hull sidewalls. Check the length of the PU tube to the temperature sensor and adjust accordingly. Insert the Simple Penetrator into the PU tube.
6. Assemble *TOP-211-Sensor_mount_cond_01* and *TOP-211-Sensor_mount_cond_02* and mount to the bottom hull after inserting the conductivity probe.
7. Assemble *TOP-211-Sensor_mount_temp_01* and *TOP-211-Sensor_mount_temp_02* and mount to the bottom hull after inserting the temperature sensor.
8. Mount part *TOP-211-Nose*
9. Cut two pieces of PU foam (P22) of 70 mm and glue into the ballast slots on the sides of the bottom hull.
10. Mount part *TOP-253-Fork* to *TOP-253-Ballast_holder* and install on the bottom hull.

### Electronics

1. Solder components to *PCB_01* and *PCB_02* using the supplied schematics for reference.
2. Cut the trace between VIN and VUSB on the back of the Teensy 4.1 (P23) board to allow both USB and battery power [14].
3. Create a 180 mm wire harness connected to connector P24 on PCB_01 and connector P25 using the schematics for reference. Insert P25 into part *TOP-280-Connector_housing* to secure the contacts.
  a. We used a braided sleeve and heat shrink tubing to organize the harness.
4. Cut a Ø7 mm carbon tube (P26) to a length of 40 mm and use it to mount part *TOP-211-Electronics_frame_main_01* to *TOP-211-Electronics_frame_top* already installed on the top endcap.
5. Cut a Ø3 mm carbon rod to a length of 90 mm and glue it into *TOP-211-Electronics_frame_main_01* in the position shown in the CAD assembly.
6. Install *PCB_01* on the part *TOP-211-Electronics_frame_main_01*.
7. Install leak sensor probes (P27) in the bottom endcap using the adhesive supplied with the probes.
8. Install *PCB_02* on the part *TOP-211-Electronics_frame_bottom*.
9. Install the appropriate connectors on all wires and connect them to the PCB’s.
10. Secure wires to the available mounting points around the frame to make sure no wires will suffer damage during canister assembly and disassembly.
11. Install two 50 mm carbon rods in part *TOP-211-Electronics_frame_main_02* for securing the battery.

### Arms

1. Cut paracord (P28) to a length of 1200 mm. Tie one end to the part *TOP-251-Wire_mount*.
2. Tread the other end through the loops in the top hull, from bottom to top.
3. If a winch is to be used, install the coupler *TOP-251-Wire_coupler* and tie a knot to prevent it from moving along the cord.
4. Run the cord through the arms *TOP-251-Arm_500mm* with the buoyancy foam *TOP-251-Buoyancy* and the part *TOP-251-Fork* already installed.
5. Repeat the procedure above in reverse for the other side.
6. Install PU foam through the opening in part *TOP-211-Top_hull_antenna_mount* and fasten it to the arm using cable ties.
7. Install small blocks of PU foam to the arms on either side of the part *TOP-251-For*k with cable ties to provide the releasing force for actuation. See Fig. 21.

### Battery

1. Spot weld four balanced cells (P29) in series in a flat configuration with alternating polarity
2. Insert the cells into the holders *TOP-260-Batteri_frame* and *TOP-260-Batteri_frame_mirror*.
3. Screw the part *TOP-260-Battery_frame_bottom* to the two cell holders and mount the BMS (P30) over the designed pins.
4. Heat and compress the pins to lock the BMS in place.
5. Solder connections to the BMS in a common port configuration and secure the main battery leads to the cell holders using cable ties. Solder a female XT30 (P31) connector to the battery leads.
6. Cover the battery pack in heat shrink tubing (P32) and heat until the tubing shrinks tight around the pack.

### UAV

1. Cut the carbon fibre plates (P33) in the specified shape, along with the various Ø7 mm carbon fibre tubes and Ø3 mm carbon fibre rods.
2. The tube and rod assemblies are designed for compression fits but depending on printer tolerances some adhesive might be necessary.
3. The nylon webbing (P34) must be sewn to the correct size. Test fit with the assembled profiler and cut length accordingly.
4. Attach a 3 m long paracord to the hook and coil it on the spool.

## Operation instructions

For safe operations, we recommend deploying the UAV from land. Greenland fjords are often about 2–5 km across in the inner glacier covered parts and launching the UAV at a safe distance above tsunami (from glacier calving) water levels a 1 km range is often sufficient to reach the subglacial discharge plume.

### Pre deployment

**Fig. 20:**
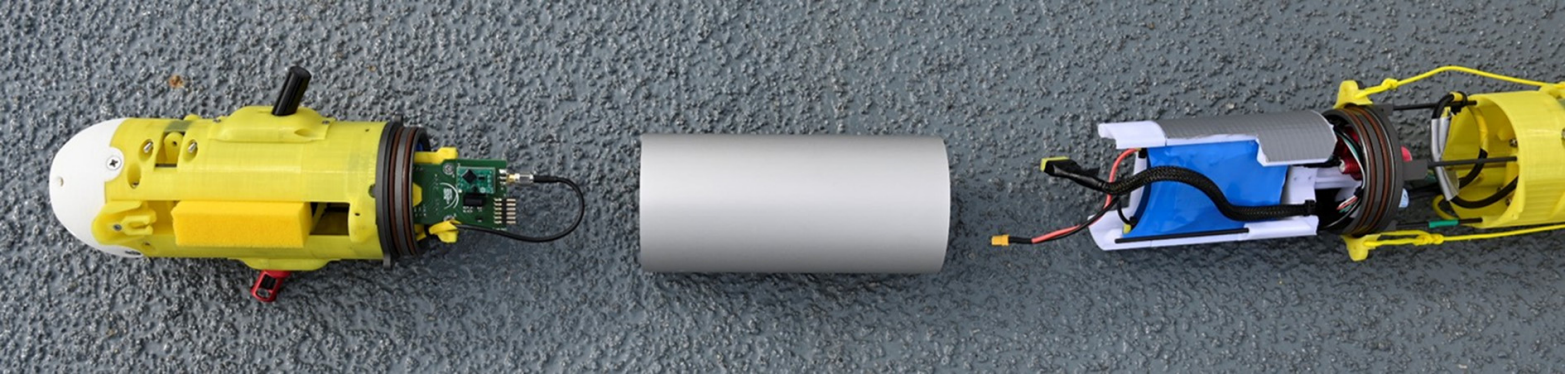
The disassembled profiler before preparing for deployment. From left to right: Bottom hull, pressure hull and top hull. Photo courtesy of Peter Bondo Christensen.

At this stage, the hull is assumed to be disassembled as shown in Fig. 20.

To prepare for deployment:

1. Charge the profiler battery to 16.8 V.
2. Insert SD card in the profiler if not already inserted.
3. Place ballast in bottom hull by compressing the PU foam, inserting ballast and closing the bottom cover. Hold tight while inserting a PCL rod through the locking levers and repeat for the other ballast.
4. While holding the ballast tight use M3 locking nuts on the PCL rod to secure the ballast in place. See Fig. 21 for reference. The ballast actuator is identical to the pick-up arm actuator.

**Fig. 21:**
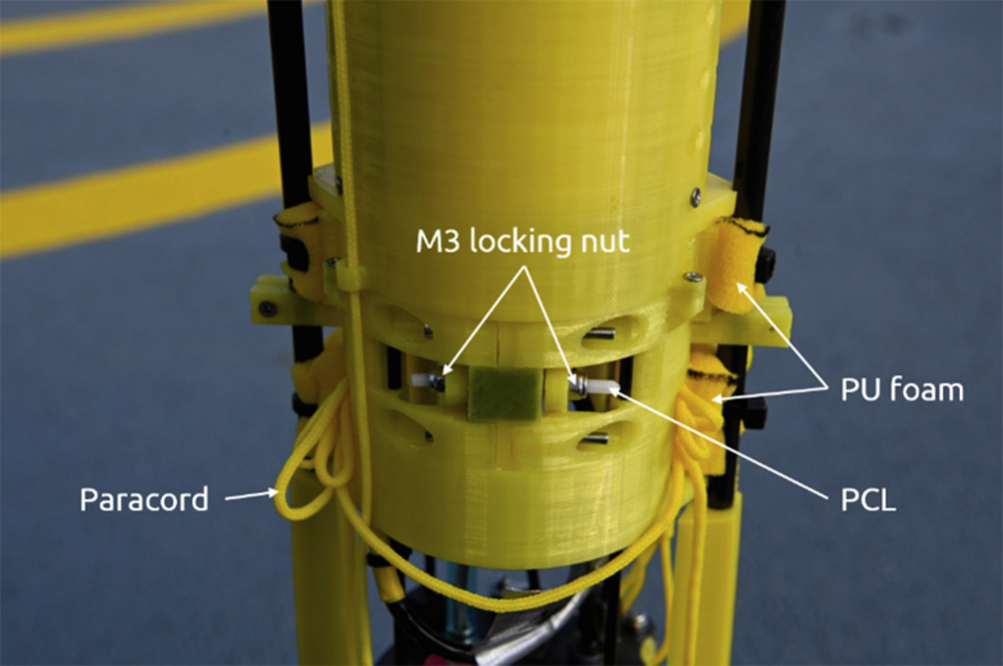
Overview of the loaded pick-up arms and actuator. Photo courtesy of Peter Bondo Christensen.
5. Load the pick-up arms by tightening the paracord and bundling all excess cord. While holding these bundles to the sides of the top hull place the arms along the sides of the top hull. Make sure the forks are inserted into the holes next to the heating element. The foam blocks should keep the cord bundles in place.
6. Hold the arms in place while inserting a PCL rod into the heating element and locking levers. Secure with two M3 locking nuts on the PCL rod. See Fig. 21 for reference.

### Power on and assembling

1. Remove the vent plug to allow air to escape during assembly.
2. Insert the charged battery into the slot on the electronics frame *TOP-211-Electronics_frame_main_01*.
3. Insert the spacer *TOP-211-Electronics_frame_main_02* to secure the battery.
4. Slide the canister (P35) over the assembly and the top endcap. It should snap into place if the two O-rings are properly lubricated.
5. Connect the cable harness to *PCB_02* on the bottom hull.
6. Connect the ground station *PCB_04* to a PC and open a serial monitor on the connected port.
7. Power on the ARC-TOP by connecting the battery to *PCB_01*.
8. Check the serial monitor on the ground station and the indicator LED on the profiler. Proceed with assembly if no error is indicated.
9. Slide the bottom endcap into the canister using the guide pin to align the assemblies. It should snap into place if the O-rings are properly lubricated.
10. Secure the pressure hull by installing the M4 threaded rods with locking nuts.
11. If possible, a vacuum test should be conducted to test for any leaks in the system. We used an Ametek T-975-CPF pump and HPC40 sensor [30], [31]. Cheaper options like the Mityvac Hand Operated Vacuum Pump [32] should suffice.
12. Close vent plug.

### Prepare for dive

1. Set dive parameters by sending the appropriate command (see *Naming_and_BOM.xlsx* for command API).
2. Prepare hook on the UAV by inserting the spool into the bracket and mounting the hook.
3. Mount the profiler on the UAV.
4. Send arm command to profiler and check for correct status response.

### Deploy

1. Continuously monitor ground station for correct status response from the profiler. Abort deployment if error detected.
2. Fly UAV to deployment position and deploy at a height of max 1 m above sea level.
3. Depending on UAV flight time, either hover until retrieval or return to home position.

### Retrieval

1. Monitor ground station for profiler surface message. The broadcasted message contains the position of the profiler.
2. When a message is received, fly the UAV to the profiler position.
3. When the profiler is identified deploy the hook.
4. Lower the hook into the water by slowly decreasing UAV altitude.
5. Drag the hook through the water until it catches the profiler pick-up arms.
6. Increase the UAV altitude until the profiler is clear of the water.
7. Pilot the UAV to the home position being mindful of the pendulum effect of the suspended mass of the profiler.
8. Gently settle the profiler on the ground before landing the UAV.

### Disarm and securing

1. Send disarm command to the profiler.
2. If no error is indicated, rinse the profiler in fresh water to prevent corrosion.
3. Clean the actuator heating elements by sending the appropriate reload commands and inserting a Ø3 mm carbon rod into the element. This will remove any leftover PCL and prepare for the next deployment.
4. Thoroughly dry the profiler and open the canister after removing the vent plug.
  a. Remember to compress the PU foam to extract the absorbed water.
5. Unplug the battery from *PCB_01* and transfer the SD card from *PCB_01* to a suitable reader to download data to a computer.

## Validation and characterization

**Fig. 22:**
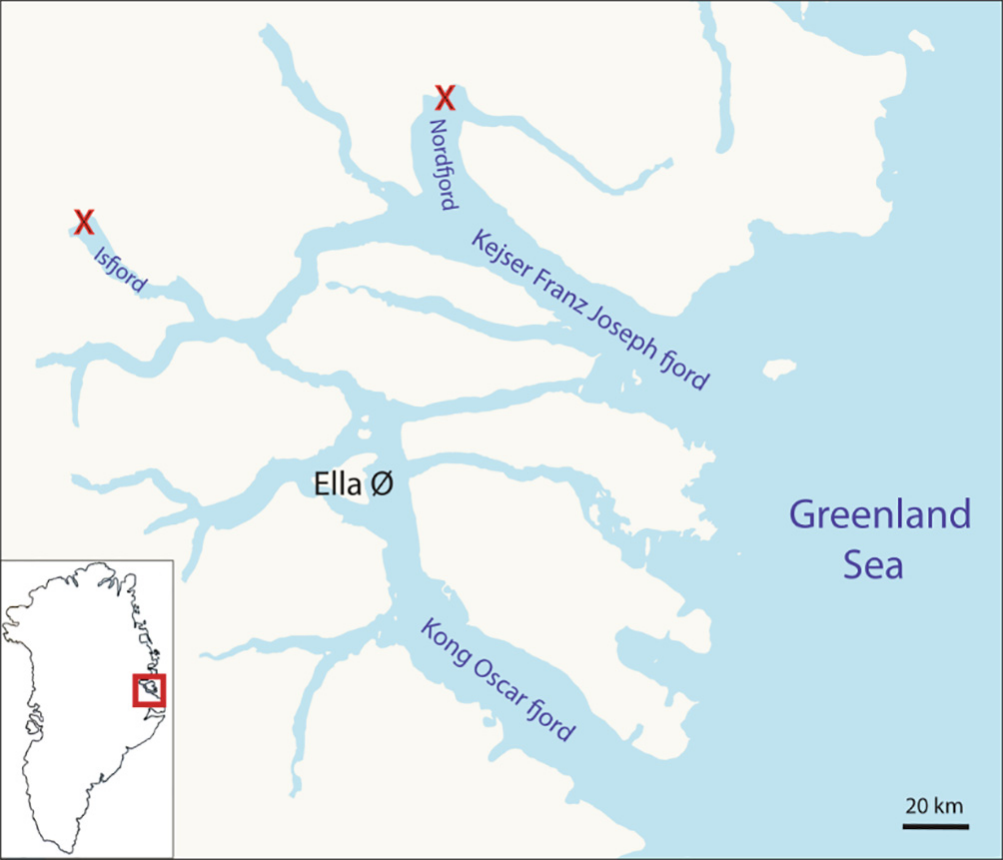
Test locations in NE Greenland in Isfjord and Nordfjord marked with X.

The ARC-TOP profiler was tested during August of 2021 on a cruise in NE Greenland. Testing was intensified at the Waltershausen Glacier in Nordfjord (73.5°N, 24.11°W) and at the Gerard de Geer Glacier in Isfjord (73.3°N, 27.2°W). See Fig. 22.

### Operational feasibility

Testing the operational feasibility of the concept was done by conducting tethered dives to depths of up to 200 m where the profiler was free to move in the vertical axis along the tether. During these tests the ARC-TOP was deployed by hand and used its buoyancy engine to return to the surface. The profiler successfully demonstrated autonomous operation of the sensor system, buoyancy engine, pick-up arms and radio communications.

**Fig. 23:**
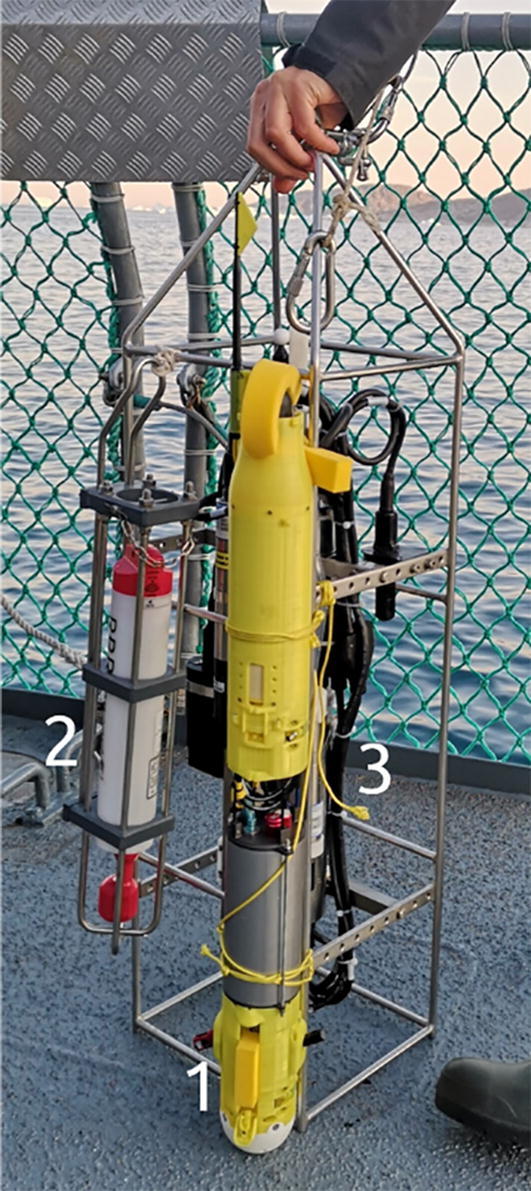
Simultaneous testing of CTD instruments. ARC-TOP (1), RBRconcerto (2) and Seabird SBE 19plus (3 behind ARC-TOP).

**Fig. 24:**
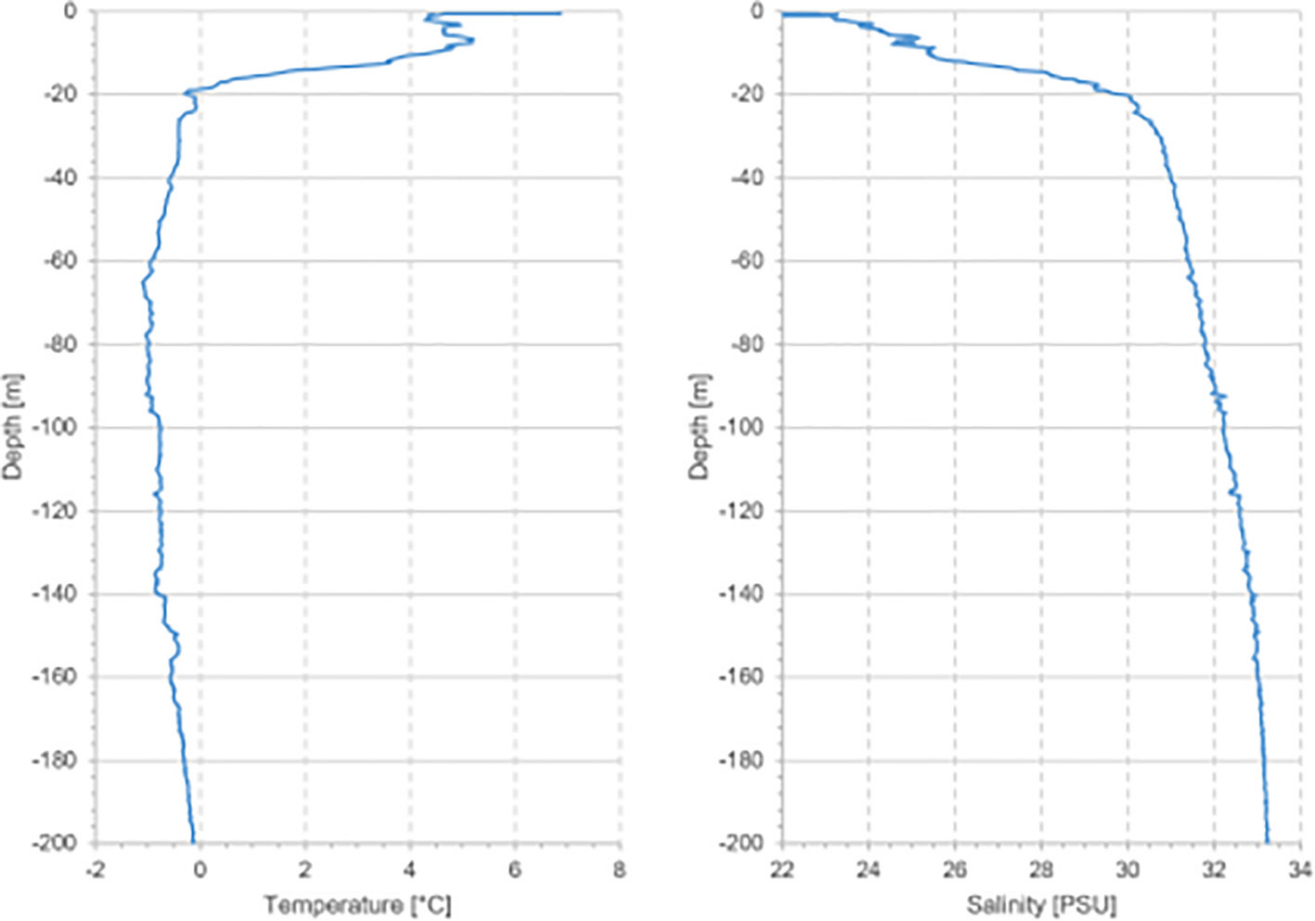
Temperature (left) and salinity (right) measurements by ARC-TOP approximately 200 m from Gerard de Geer Glacier in Isfjord (left marking in Fig. 22).

To further validate the sensor system we conducted tests in concert with commercially available CTD instruments. The ARC-TOP was tested with an RBR*concerto* and a Seabird SBE 19plus in the configuration shown in Fig. 23. No significant difference were found between the calibrated temperature (paired *t*-test, p = 0.27, α = 0.05) and salinity readings (paired *t*-test, p = 0.086, α = 0.05) of the ARC-TOP and SBE 19plus instruments. An example of recorded data in close proximity to glacier termini is presented in Fig. 24.

### Remote deployment and retrieval

**Fig. 25:**
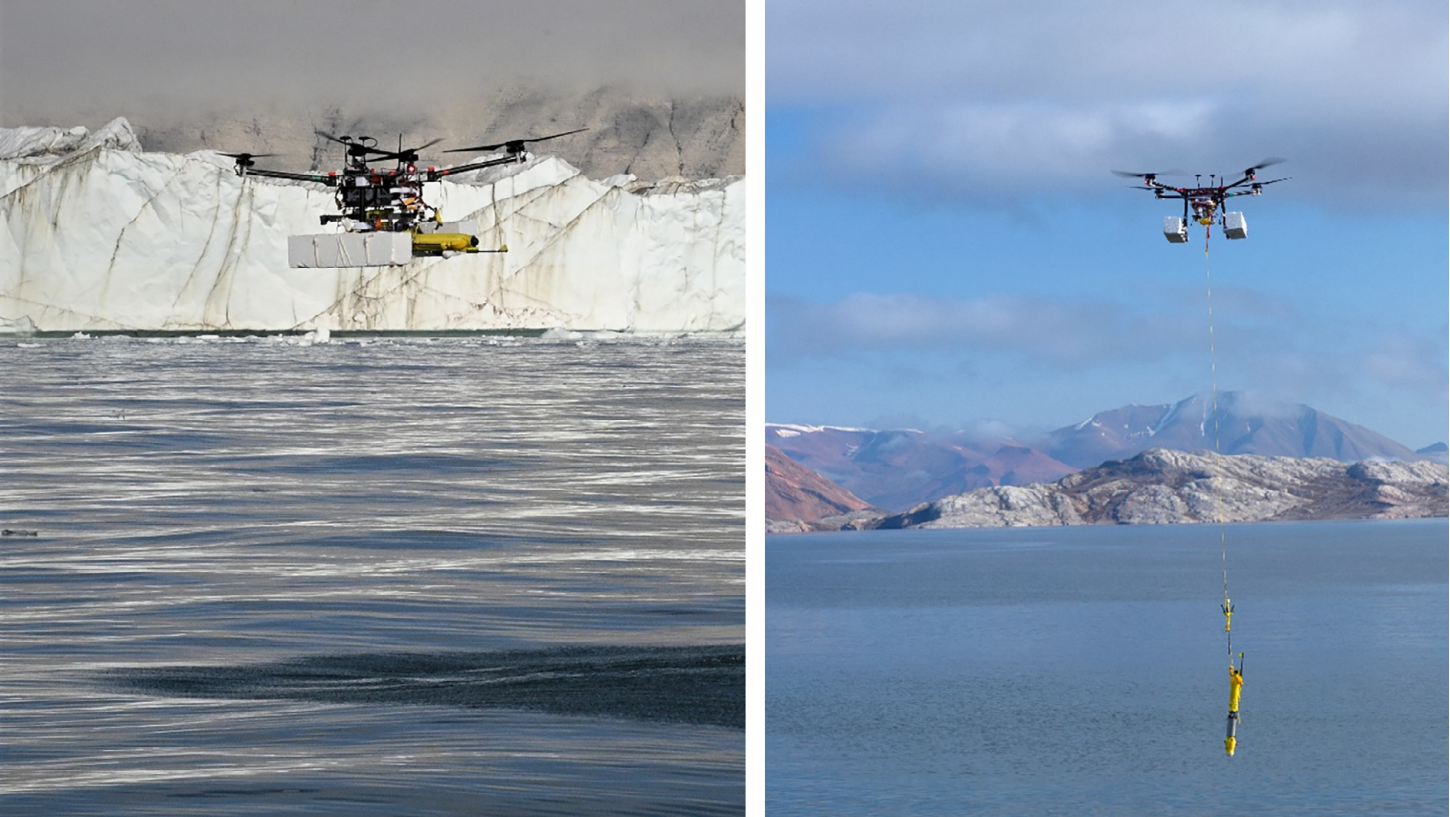
Profiler delivery just prior to deployment (left), photo courtesy of Peter Bondo Christensen., and profiler retrieval after deployment (right), photo courtesy of Nils Risgaard-Petersen.

Remote deployments and retrievals were carried out near glacier termini as shown in Fig. 25. The testing took place from marine vessels in calm weather and within line-of-sight of the measurement location. GPS data showed a maximum distance between operator and the ARC-TOP of approx. 1 km during testing. The profiler was able to correctly detect deployment and autonomously operate nominally.

### Performance assessment

The general performance assessment with reference to the listed requirements can be seen in Table 1.

During remote deployment the number of samples pr. vertical meter was found to be 3.51 m^−1^ (σ = 0.1 m^−1^), well within the requirement listed in Table 1. The dive velocity is 0.48 m/s (σ = 0.01 m/s).

The used Atlas Mini Conductivity Probe K 1.0 and EZO Conductivity circuit have been calibrated in-house after the cruise to determine whether the rated accuracy is governed by a poor calibration or general sensor performance. A two-point calibration was conducted in a small tank (150 L) with known temperature and salinity. A Guildline 8410A Portasal salinometer [33] was used as reference. Continuous circulation was ensured during sampling. A resulting expanded uncertainty of 0.07 mS/cm (α = 0.05) was calculated from a simple linear calibration curve at a measurement of 31 × 10^3^ mS/cm. This result is significantly better than the manufacturer stated accuracy of ± 1 % i.e. 0.31 mS/cm [34], but not all sources of uncertainty have been identified and the total temperature and pressure range have not been investigated. This only serves as an indication that these cost-effective sensors can be used successfully with proper calibration.

During deployment near glacier termini high lateral displacement was observed. This negatively affects the operational success rate as the profiler can become trapped under sea ice or the glacier itself.

Handling the instrument proved possible but time-consuming as the pressure hull needs disassembly and assembly between deployments. This leads to increased wear of the sealing surfaces and higher risks of leakage, but recommendations to alleviate this are presented in the next section.

### Future improvements

The ARC-TOP concept have been shown to be feasible in producing measurements in remote or hazardous areas. Areas of the design were identified for further development during testing to improve the system performance and usability.

#### Power and data transmission.

A revised power and data transmission system will improve the ease of use and reliability of the system. Key areas of improvement are:

1. External power switch enabling cycling the power without the need to disassemble the profiler.
2. External charging port to allow recharging the battery while it is installed in the profiler.
3. Reduce the capacity of the battery to reduce the overall size. The current battery is highly oversized for the described mission.
4. Software for wireless data transmission to ground station with error detection.
  a. The current electronics are fully capable of this without modifications.
5. Increased transmission range by optimizing antenna performance.
6. Increased UAV range by optimizing UAV power system and signal transmission

#### Design

The general design of the concept can be improved in the following areas:

1. Weight and size can be reduced further by adopting a design not made for repeated disassembly. This will be possible by making the changes to the power system outlined above. This will potentially reduce the electronic complexity by eliminating the need for two PCB’s in favor of a single PCB design.
2. Both actuators are sensitive to the flexibility and tolerances of its components. A machined assembly will mitigate this and thus improve reliability and handling.
3. External cabling on the ARC-TOP is a major risk of leak due to damage during assembly and usage. Improved cable insulator quality and protection should be considered as well as reducing the cabling length.
4. Reduce PU tubing diameter connected to Simple Penetrators for increased flexibility and ease of assembly.
5. Increasing visibility while the profiler is at the surface for easy visual detection in the event of GPS or signal transmission failure.

#### Production

1. Changing currently 3D-printed parts to injection-molded parts will significantly increase the strength and durability of the instrument while simplifying assembly. This will however increase costs at low production volumes.

## Ethics statements

Not relevant.

## CRediT authorship contribution statement

**Ebbe Poulsen:** Writing – original draft, Project administration, Investigation, Software, Formal analysis. **Mathias Eggertsen:** Validation, Investigation, Formal analysis, Project administration. **Erik H. Jepsen:** Validation, Investigation, Project administration. **Claus Melvad:** Supervision, Funding acquisition, Conceptualization, Writing – review & editing. **Søren Rysgaard:** Investigation, Writing – review & editing, Supervision, Funding acquisition, Conceptualization.

## Declaration of Competing Interest

The authors declare that they have no known competing financial interests or personal relationships that could have appeared to influence the work reported in this paper.
